# Biotic response networks are an important organizer of the transcriptome in wild *Arabidopsis thaliana* populations

**DOI:** 10.1073/pnas.2611539123

**Published:** 2026-10-19

**Authors:** Ana Paula Leite Montalvão, Kevin D. Murray, Ilja Bezrukov, Natalie Betz, Lucas P. Henry, Paloma Duran, Patrick Boppert, Martina Kolb, TEAM PATHOCOM, Fabrice Roux, Joy Bergelson, Detlef Weigel, Wei Yuan

**Affiliations:** ^a^ Department of Molecular Biology, Max Planck Institute for Biology Tübingen, Tübingen 72076, Germany; ^b^ Department of Biology, New York University, New York, NY 10003; ^c^ Center for Genomics and Systems Biology, New York University, New York, NY 10003; ^d^ Laboratoire des Interactions Plantes-Microrganismes, CNRS, Institut national de recherche pour l‘agriculture, l‘alimentation et l‘environnement, Université de Toulouse, Castanet Tolosan 31326, France; ^e^ Institute for Bioinformatics and Medical Informatics, University of Tübingen, Tübingen 72076, Germany

**Keywords:** coexpression networks, host–microbiome interactions, molecular ecology, ecological genomics, ecological transcriptomics

## Abstract

Extensive laboratory experimentation has revealed conserved molecular pathways controlling growth and stress responses in plants, yet how these programs operate in natural settings remains poorly understood. We have investigated the organization of the transcriptome in wild populations of *Arabidopsis thaliana* by sampling plants across 60 natural sites in Europe and North America over two seasons. Transcriptomes varied extensively among individuals and were only weakly structured by geography or season. No groups of plants stood out due to clear biotic or abiotic stress signatures known from the laboratory. While only a small portion of transcriptome variance was explained by disease symptoms or environmental and phenotypic variables, infection-associated signals accounted for the largest share of the explainable fraction. Biotic-response networks identified in controlled laboratory conditions were well conserved in wild transcriptomes, while other networks, including abiotic-response networks were substantially reorganized in wild plants. Together, these results indicate that while certain transcriptional modules are easily reidentified when moving from the wild to controlled conditions, many regulatory relationships differ in these two contexts, highlighting how ecological complexity shapes transcriptome organization in natural populations.

## Significance

In nature, plants experience a complex mixture of stresses, yet most of our understanding of plant gene regulation comes from the application of single stresses to genetically uniform individuals. We profiled transcriptomes of wild *Arabidopsis thaliana* plants across 60 natural sites. Although most transcriptome variation remained unexplained by measurements of microbial infection, weather, and visual plant traits, infection-associated signals consistently accounted for more variance than abiotic variables. Comparing wild and laboratory transcriptomes revealed that biotic-response networks are well preserved while abiotic-response networks are substantially reorganized in nature. These findings suggest that laboratory experiments, despite their power to identify causal pathways, have explored only a fraction of the transcriptional states plants adopt in nature.

Over the past decades, controlled laboratory experiments have revealed genetic and molecular pathways controlling development, metabolism, stress physiology, and immunity. While insights from these studies have helped to improve the performance of crop plants or to develop treatments for human disease, translation from the bench to the field or bedside often fails (1–6). One important reason is that we only have a limited understanding of how organisms respond to highly dynamic and complex environments. In the wild, organisms experience overlapping abiotic and biotic stresses throughout their life cycle, and responses to new challenges are shaped by developmental state, life history, and interactions with other species (7). In contrast, laboratory studies typically apply defined perturbations to genetically identical individuals in uniform environments, thereby maximizing the chances of identifying causal effects. Even when laboratory designs attempt to mimic complex environments, it is difficult to reproduce the combinations, sequences, and fluctuations of stressors encountered in nature. Because abiotic and biotic cues can also differ in their spatial and temporal structure, we do not know whether and how easily the principles governing organismal responses generalize from the laboratory to the wild. Understanding how coexpression networks are reorganized in such highly dynamic and complex surroundings is thus essential for bridging laboratory and field biology (8).

Because plants are sessile, variation in their local environment over the life cycle is more easily recorded than for most animals. Among plants, *Arabidopsis thaliana* stands out due to the wealth of laboratory studies that have probed its responses to many different environmental stressors. This in turn has led to the identification of many molecular regulators that shape these responses, even though it remains difficult to predict how important these factors are under natural conditions. A prime example comes from the study of *FLC* and *FRIGIDA*, which together explain much of the flowering time variation among *A. thaliana* strains in the greenhouse, but which have much more limited effects when plants are grown outdoors in common gardens or directly in their native habitats at the time of year that matches the phenology of natural cohorts at the respective location (2, 9–11).

Transcriptome profiling provides a less observer-biased, high-dimensional molecular phenotype that can capture responses beyond preselected, visually scored traits. The study of transcriptome dynamics in the field has a considerable history, although the overall number of such studies is still limited (12–22). Most outdoor transcriptomic studies deliberately reduce microenvironmental and life-history heterogeneity and thereby partially disentangle genotype from local ecology by, for example, using common gardens or sampling native plants with a design that minimizes interannual variation. It therefore remains unclear how transcriptome organization is structured in wild individuals, where genetic background is nested within and shaped alongside local environments and phenotypes.

Here, we examined transcriptome organization directly in wild *A. thaliana* populations, asking how the organization of transcriptional networks in the wild compares to such networks in the laboratory. Although environmental and phenotypic factors explained little total variance, infection-associated variables accounted for more variation in wild transcriptomes than abiotic factors. This aligns with wild transcriptomes closely reflecting the activity of laboratory-defined biotic response networks, whereas control and abiotic response networks are substantially reorganized. Our findings suggest that while core functional modules identified in laboratory experiments remain recognizable in natural populations, their organization and activity differ, and that laboratory studies have likely only explored a small portion of all possible transcriptome states.

## Results

### Overall Patterns of Transcriptome Variation in Wild Samples.

**Fig. 1.**
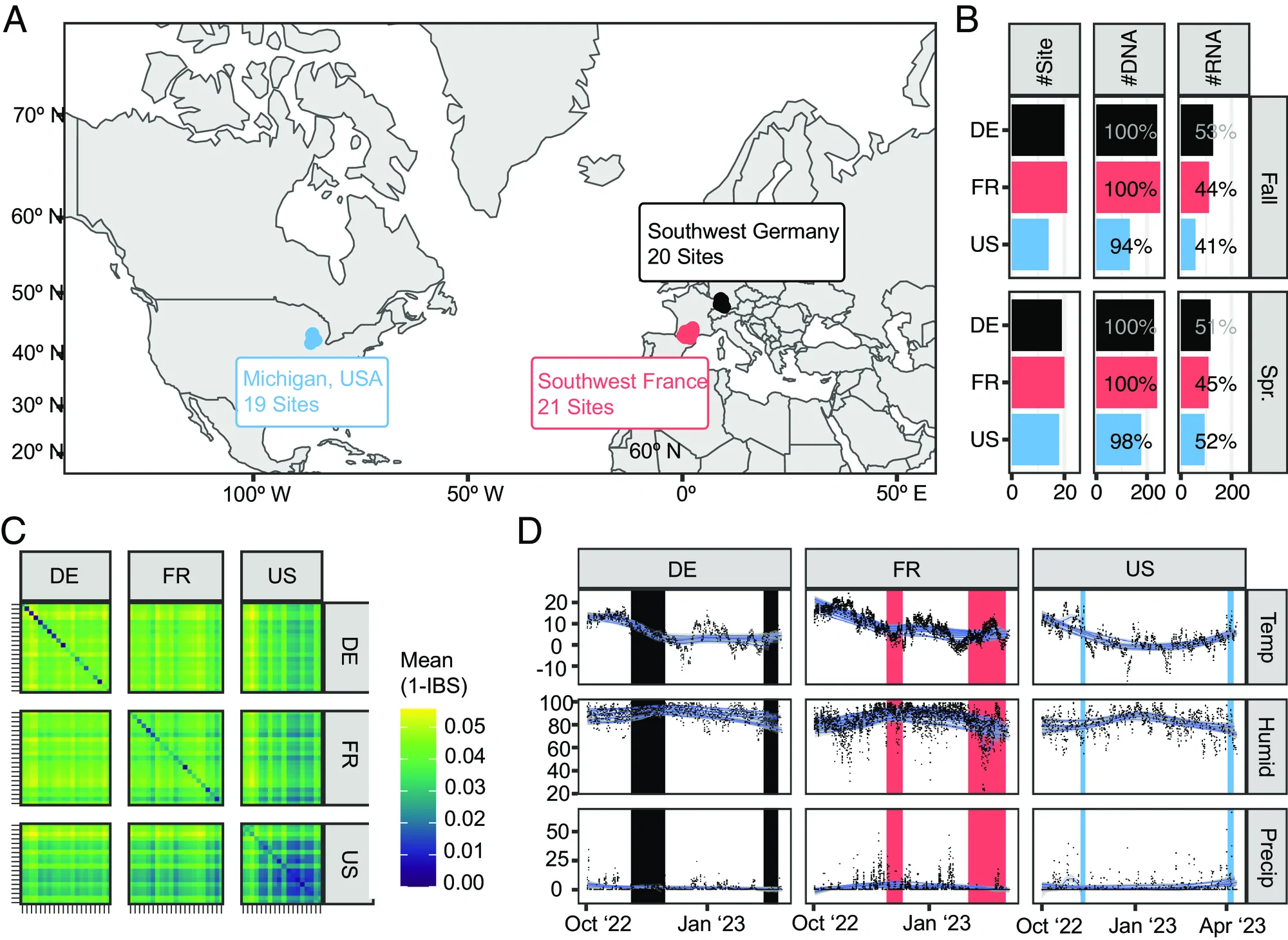
Summary of sample collection. (*A*) Samples were collected from 60 sites across southwest Germany (DE, 20 sites in October to November 2022, 19 sites in February to March 2023), southeastern France (FR, 21 sites in November to December 2022; 20 sites in January to March 2023), and Michigan (US, 14 sites in October to November 2022, 18 sites in April 2023). (*B*) Number of samples where DNA- and RNA-sequencing results passed our quality thresholds. *X*-axis: number of samples; percentages: proportion of all plants collected. (*C*) Genetic diversity (measured as one minus Identity-by-State [IBS]) within and between countries. (*D*) Average daily temperature (°C), humidity (% RH), and total daily precipitation (mm) across the 2022–2023 growing season, as recorded by nearby weather stations. Points are days per site, and blue lines with gray bars are GAM-smoothed running means. Colored vertical bars represent the sampling periods in each country.

**Fig. 2.**
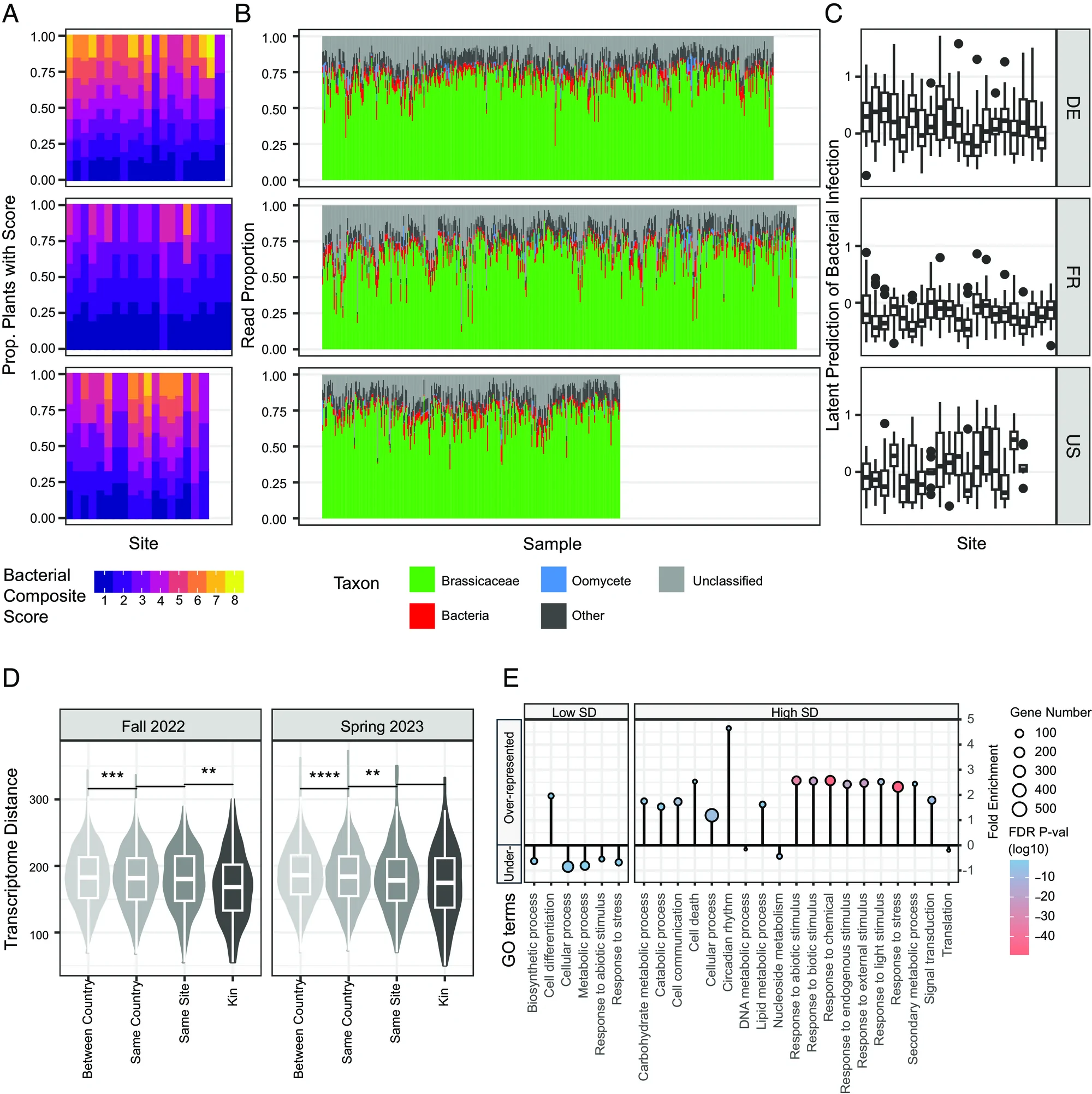
Genetic, phenotypic, and transcriptome variation. (*A*) Composite bacterial scores, which are the sums of all symptoms suggestive of bacterial infection. Variation exists among both sites and countries, with the fewest symptoms in France. (*B*) Microbial load and composition. Samples are ordered by site and season. The fraction of microbial reads in the DNA samples, a proxy of microbial load, varies from under 5 to 75%. The taxa with the most assigned reads are *Pseudomonas*, *Sphingomonas*, *Hyaloperonospora,* and *Albugo*. (*C*) Bacterial infections, as estimated with confirmatory factor analysis. Country labels in C also apply to *A* and *B*. (*D*) Distribution of pairwise transcriptome distances from individuals grouped by geographic location (Between Countries, within the Same Country but between sites, and within the Same Site) and genetic similarity (Kin, pairs of samples within sites with coefficients of relatedness ≥0.125). Significant differences between pairs indicated with one or more asterisks (Wilcoxon test). (*E*) Under- and overrepresented GO terms for low-SD and high-SD genes. Circle size indicates number of genes in category, and color represents the FDR-corrected *P*-value, with red representing highly significant associations.

We sampled up to 12 *A. thaliana* plants from 60 sites across Germany, France, and the United States in fall 2022 and spring 2023 (Fig. 1 *A* and *B*; https://doi.org/10.6084/m9.figshare.31638136). For each plant, we measured microbial abundance and diversity, field scores of disease and developmental state, along with recent precipitation, temperature, and humidity, to capture seasonal variation in factors that may shape the transcriptome (Figs. 1 *C* and *D* and 2 *A* and *B*, *SI Appendix*, Figs. S1 and S2, and Datasets S1 and S2). The time and date of sampling were also recorded. After washing with sterile distilled water to remove larger soil particles, DNA and RNA were extracted from each plant. Shotgun sequencing of DNA captured both epiphytic and endophytic microbial load together with plant genotype (23) (https://doi.org/10.6084/m9.figshare.31638136), but for RNA sequencing we only considered reads that mapped to the *A. thaliana* reference genome (https://doi.org/10.6084/m9.figshare.31638136).

From 1,269 plants collected, we obtained 616 transcriptome samples, 1,267 metagenome profiles, and 1,092 host genotype calls, with 616 samples having all data types (Fig. 1*B*). The phenology of plants broadly aligned with the known life history of *A. thaliana* and its pathobiota (24), with plants being larger, more likely to be flowering, and having more signs of disease in spring than in fall (25, 26) (*SI Appendix*, Fig. S2 *B*–*D*). Symptoms associated with bacterial, fungal, or oomycete disease were not strongly correlated (*SI Appendix*, Figs. S2 *C* and *D* and S3*B*), with the exception of chlorosis and necrosis, both indicators of possible bacterial infection, among other causes (24, 27–29). A composite bacterial infection score was calculated as the sum of chlorosis and necrosis scores for each individual (Fig. 2*A*). Patterns of genetic relatedness within and between sites largely followed previous observations, with US sites being genetically most uniform, and French sites being most diverse (30–32) (Fig. 1*C* and *SI Appendix*, Figs. S1*C* and S2*A*). Microbial load and composition differed considerably among plants, even within sites (Fig. 2*B* and *SI Appendix*, Fig. S1*B*).

Variation in microbial load, defined as the ratio of DNA sequencing reads assigned to microbial versus *A. thaliana* genomes, was larger within than between countries, with country, site, and season explaining 3%, 19%, and 9% of variation in total nonplant load, respectively (ANOVA, *P* < 1e−10). Visible signs of disease varied in their correlation with microbial load: correlations were relatively strong for *Albugo* spp. (*r* = 0.75; *P* < 2e−16), a major pathogen of *A. thaliana* (33, 34), weaker for *Hyaloperonospora* spp. (*r* = 0.22, *P* < 8e−15), likely due to its longer asymptomatic phase, and weakest for bacterial pathogens (*r* = 0.14, *P* < 3e−6; *SI Appendix*, Fig. S3*B*), likely due to both asymptomatic infections (24, 35) and difficulties in scoring bacterial disease symptoms on small plants. We therefore performed Confirmatory Factor Analysis to synthesize latent estimates of infection from these multiple predictors of uncertain accuracy for use in subsequent analyses (Fig. 2*C* and *SI Appendix*, Fig. S3).

Exploratory analyses with principal component analysis (PCA) of transcriptomes and microbiomes showed data points to be widely dispersed, without clear clusters of subsets of samples (*SI Appendix*, Fig. S1 *A* and *B*). We had expected the median transcriptome distance to be lower within sites or countries and within each season, but this was only marginally the case (Fig. 2*D*). Similarly, the transcriptome distances among pairs of plants with recent kinship (estimated relatedness ≥0.125, i.e., first cousin or closer) were only marginally lower than for unrelated pairs from the same site and season (Fig. 2*D*). These results likely reflect the highly individualized life histories even among closely related plants within a single site.

Because plants were sampled at different times of day, we tested for residual effects of hours after sunrise while controlling for country and season. Only 51 of 18,452 genes were significant after Benjamini–Hochberg correction, with very small effect sizes. This did not include any of 16 canonical core clock genes. Thus, after accounting for geography and season, sampling time-of-day explained little variation.

To better understand the variance distribution of the wild transcriptome, we identified the 5% most and least variable genes based on the SD of normalized expression across all samples (high- and low-SD genes; *SI Appendix*, Fig. S4*A* and Dataset S3). Because SD was calculated on variance-stabilized and technically corrected expression values, it reflects residual among-sample variation after reducing the mean-variance dependence typical of RNA-seq count data. While low-SD genes were exclusively at the extreme ends of mean expression levels, high-SD genes tended to be expressed at intermediate levels, consistent with the expectation that one has the greatest power to detect variation for genes that can easily vary in both directions from their mean (*SI Appendix*, Fig. S4*B*). High-SD genes were strongly enriched for GO categories related to response to endogenous and external stimuli but depleted for housekeeping functions, while this trend was reversed for low-SD genes (Fig. 2*E*). Due to the study design, we cannot rule out that expression variation can at least in part be attributed to higher genetic and/or regulatory diversity in these genes. A comparison of mean pairwise genetic distance (*π*) among all samples at each gene (±1 kb) found no significant difference between low- and high-SD genes (Wilcoxon test *W* = 421,636, *P* = 0.2374), nor between low-SD genes and 1,000 randomly selected genes (*W* = 429,916, *P* = 0.5053). High-SD genes, though not significant (*W* = 456,822, *P* = 0.05661), showed a greater trend toward having higher genetic diversity. Consistent with a possible role of local adaptation, most high- and low-SD genes were unique to a single country and season (*SI Appendix*, Fig. S4 *C* and *D*).

**Fig. 3.**
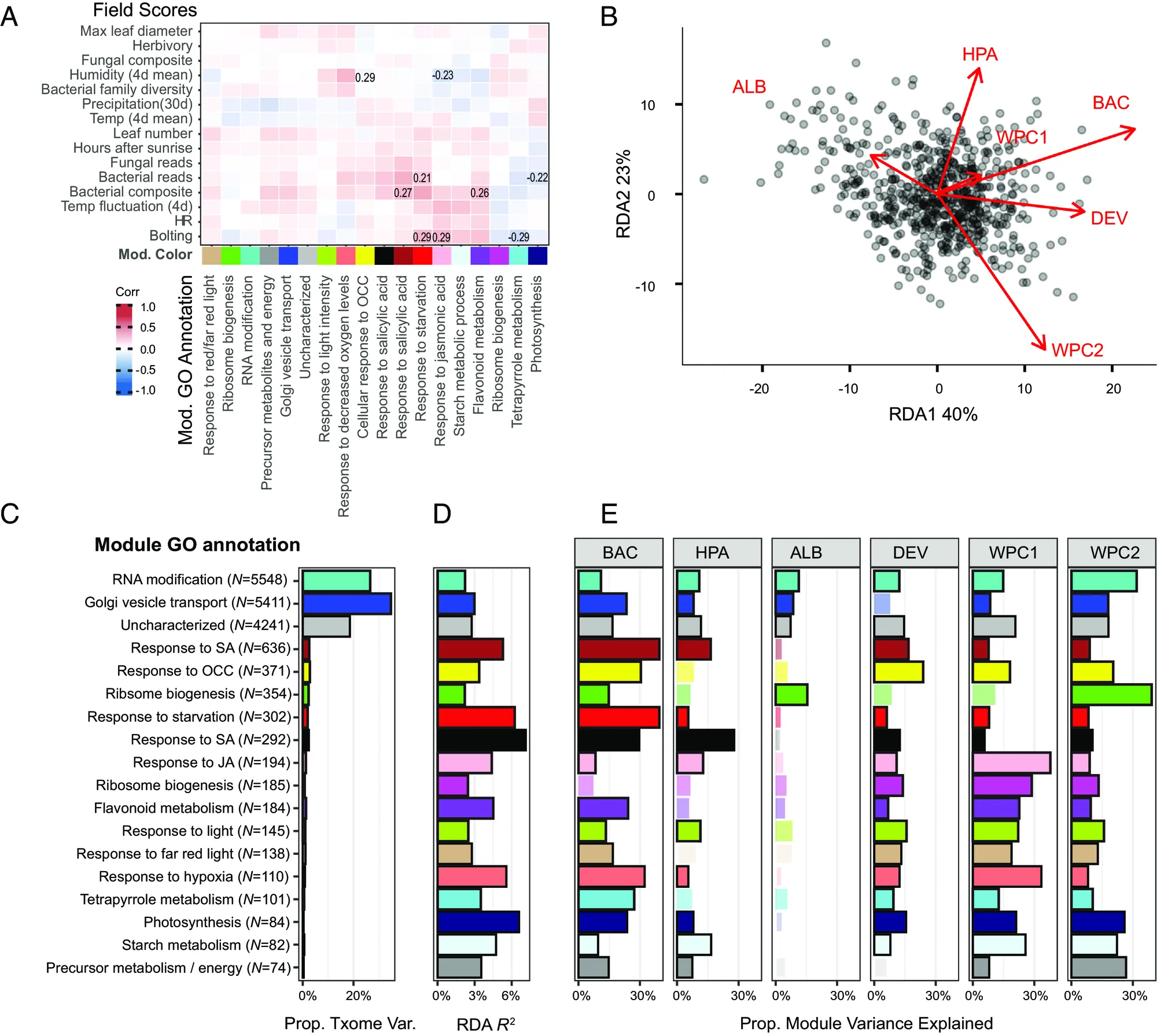
Factors explaining transcriptome variation. (*A*) Heatmap of Pearson’s *r* between WGCNA module eigengenes (MEs) and observed plant and environmental variables. Red indicates positive and blue negative correlation. Modules are labeled by top enriched GO biological process terms, together with the designated color labels (colored squares). Exact values are given for correlation coefficients >0.2 and *P* < 0.05. (*B*) RDA analysis of the global transcriptome, constraining transcriptome variation by latent factor predictions of infection by bacteria (BAC), *Hyaloperonospora* (HPA), and *Albugo* (ALB), and by latent factor predictions of developmental stage (DEV), and principal components 1 and 2 of recent weather obtained from nearby weather stations (WPC1 and WPC2). Overall, constrained axes explained 5% of total variance. (*C*) Proportion of global transcriptome variance (Prop. Txome Var.) attributable to each of 18 wild-sample-derived WGCNA modules and number of genes in each module. (*D*) Module expression variance explained in per-module RDA analyses, with the same constraining variables as in *B*. (*E*) Proportion of module expression variance uniquely explained by each constraining variable (*B*). Terms without black outlines are not statistically significant at FDR-corrected *P* < 0.05. Across all modules, mean proportions of constrained variance explained are 21% (BAC), 12% (HPA), 5% (ALB), 12% (DEV), 17% (WPC1), and 16% (WPC2). Color code for *C*–*E* as in *A*.

To examine the underlying biological mechanisms driving the observed transcriptomic variability, we turned to weighted gene co-expression network analysis (WGCNA), a powerful framework to group genes into functional modules (36). WGCNA grouped genes into 18 modules (Dataset S4), which we functionally annotated using GO enrichment (Fig. 3*A*). Most modules were associated with response to stimulus, and several others with metabolism, photosynthesis, and biogenesis. There were three very large modules, one related to RNA modification, one to Golgi transport, and one without significant GO enrichment (Dataset S5). These three modules mostly contained genes with low mean expression and low expression variance.

To determine possible drivers of correlated gene expression in each module, we asked how plant morphology, field scores, and microbial load correlated with the first principal component of gene expression in each module, i.e., the module eigengenes (MEs), which explained 15 to 45% of module variance (*SI Appendix*, Fig. S5 and Dataset S6). While most correlations were not significant, bacterial load and composite scores were positively correlated with multiple modules being associated with response to jasmonic acid (JA) or salicylic acid (SA) (*r* = 0.21 and 0.27, *P* = 0.001 and 0.025, respectively), and negatively correlated with a photosynthesis-related module (*r* = −0.22, *P* = 0.046; Fig. 3*A*). Consistent with the gene-level analysis, no WGCNA module correlated strongly with sampling time-of-day (maximum |bicor| = 0.19), and no module was enriched for circadian or diurnal terms. We therefore treated time-of-day as a nuisance variable.

### Bacterial Infection as Main Predictor of Transcriptome Variance.

To quantify the extent to which environmental and phenotypic variables explain transcriptome variation, we performed redundancy analysis (RDA) across all samples (Fig. 3*B*). We constrained transcriptome variation by latent factor estimates of plant development as well as bacterial, *Hyaloperonospora* and *Albugo* infection, and by principal components of environmental measurements (Dataset S7). We could explain only 5% of total transcriptome variance with these variables, with RDA1 and RDA2 together accounting for almost two thirds of the explained variance. This aligns with a complementary recent study, in which environmental variables also accounted for only a small fraction of transcriptome variation (19). Together, these results are consistent with many small, partially independent drivers shaping transcriptome differences between wild plants, with most variation being attributable to factors beyond our observations at the time of sampling.

To explore the factors that may drive expression variation within coexpressed gene modules, we performed RDA independently for each WGCNA module. Per-module models followed the global models above, with expression of all genes in a module constrained by latent factor estimates of infection with bacteria, *Hyaloperonospora* and *Albugo,* as well as estimates of developmental state and weather PCs. Similarly to the global transcriptome, most variation in expression remained unaccounted for, with at most 6% of variance explained (Fig. 3 *C* and *D*). Among the variables measured, latent estimates of pathogen infection explained the largest share of constrained expression variation (bacteria 21%, *Hyaloperonospora* 12%, *Albugo* 5%), followed by the first two PCs of weather data (PC1 17%, PC2 16%) and latent estimates of developmental stage (12%). Of note, infection explained most variation in modules where enrichment of GO terms such as response to JA or SA pointed to a role in immunity (Fig. 3*E*).

### Resemblance Between Wild Transcriptomes and Biotic Stress Response Networks in the Laboratory.

How might the global organization of transcriptomes differ between the wild and the laboratory? With biotic factors as important predictors of wild transcriptome variance, we were particularly interested in how laboratory-derived abiotic and biotic networks change in the wild. Employing a comparative WGCNA framework, we first built reference coexpression networks from public RNA-seq data of laboratory experiments (37), then projected gene expression values from fall and spring cohorts of wild plants onto these networks. We focused on a set of 1,007 biotic and 724 abiotic stress samples from 218 individual experiments (Dataset S8; https://doi.org/10.6084/m9.figshare.31638136). These datasets were selected to provide broad coverage of experimentally defined stress responses that could plausibly contribute to variation in the wild, while keeping biotic and abiotic perturbations separate so that their network organization could be contrasted directly with wild transcriptomes. We separately constructed biotic and abiotic WGCNs from the treatment samples, as well as an untreated network from 1,118 control samples. We used these networks for downstream analyses including network projection, comparison, and random forest predictions.

**Fig. 4.**
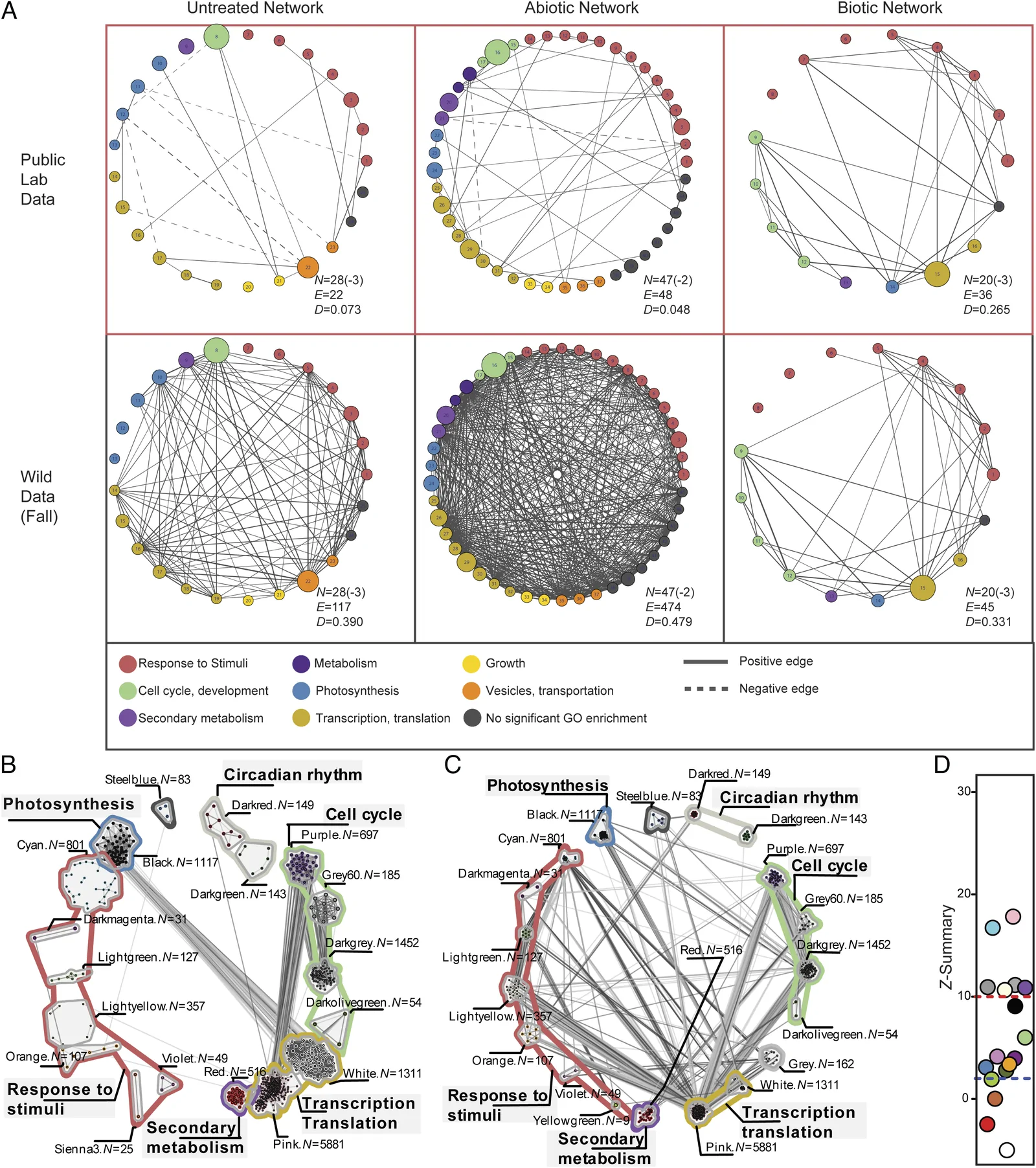
Comparative meta-analysis detects conservation and divergence of modular coexpression networks. (*A*) ME networks of public laboratory data (*Top*) and of gene expression values from the wild in fall 2022 (*Bottom*). Modules are nodes, with colors indicating GO-enrichment categories (key at the bottom). Node size indicates the number of genes in each module. Edge widths indicate strengths of connections. N: number of nodes (minus sign indicates number of circadian-rhythm-related modules); E: number of edges; D: network density. (*B*) Hub-gene network derived from the complete laboratory biotic response network (top 10% connected genes in each module). Modules are grouped by GO enrichment categories, with colored contours (color key as in *A*) around each group. Intensity of gray lines between genes indicates connection strength, with darker being stronger. (*C*) Hub-gene network with expression values from wild samples collected in fall 2022 projected onto the biotic network. (*D*) Module preservation statistics. *Z*-summary scores capture the overall conservation of module connectivity and density between the laboratory and the wild. For module annotation by color, see Dataset S11. Modules above the red dotted line are considered highly conserved, those below the blue dotted line as not conserved.

The untreated, abiotic, and biotic networks contained 28, 47, and 20 modules, respectively (Fig. 4*A*, *SI Appendix*, Fig. S6, and Datasets S9 and S10). The biotic network had a more uneven distribution of module sizes compared to the untreated and abiotic networks (Dataset S12), including one very large module with ~40% of all genes. Modules broadly mapped to the following major biological GO functions: response to stimuli, cell cycle/development, transcription/translation, photosynthesis, primary and secondary metabolism, auxin/growth, vesicle transport, and circadian rhythm (Fig. 4*A* and Dataset S11). Importantly, the networks differed substantially in their organization, with the untreated and abiotic networks being more similar to each other than either was to the biotic network. The abiotic and untreated networks had weak overall connection strength, and both positive and negative connections were similarly frequent (density 0.05/0.07, mean absolute edge weight 0.04/0.05, positive edges 56%/60%, Fig. 4*A*). The biotic network featured stronger connectivity, and more module pairs were positively correlated (density 0.27, mean absolute edge weight 0.16, positive edges 86%, Fig. 4*A*). Both abiotic and biotic networks had stronger connections between modules with related GO terms (GO assortativity, as explained in *Materials and Methods* [abiotic/biotic]: +0.18/+0.2, permutation-FDR: 0.002/0.003), compared to the untreated network (permutation-FDR = 0.246).

We projected wild transcriptomes onto the laboratory-derived networks, assigning genes to modules defined by laboratory samples and estimating eigengene values for each module. Projected module eigengene (ME) values captured on average 60% of variation in wild transcriptomes (*SI Appendix*, Fig. S7), which is comparable to the variance captured with the laboratory MEs, indicating effective dimension reduction.

Module-trait associations diverged substantially between the laboratory and the wild. In contrast to the laboratory, we did not see strong correlations with measured traits, regardless of whether we considered abiotic or biotic network projections (*SI Appendix*, Fig. S8). For example, despite ample indication of disease in the wild, both positive and negative associations of modules with field disease scores were much weaker than correlations of the same modules with microbial infections or defense hormone treatments in the laboratory. Of note, direct comparisons of wild and laboratory-raised plants have shown that the microbial loads seen after infection in the laboratory are rare in nature (38). In general, the associations in wild plants were stronger in spring, which might be due to overwinter synchronization or stronger environmental cueing with rising temperatures or lengthened photoperiods.

In the laboratory, infection-driven modules are negatively associated with growth-related functions. Strikingly, this is not the case for the wild-projected modules. As this might be due to an overall milder and more quantitative nature of the disease symptoms in the wild, we repeated the module projections using only plants with the highest and lowest disease. Associations with these disease scores in this subset were strengthened for modules related to response to stimuli and defense, but not for growth-related modules, suggesting a changed relationship between growth and defense programs in the wild.

Global network topology also differed substantially, with abiotic network modules becoming much more densely connected in the wild, with little apparent substructure (wild fall/spring [laboratory]: density 0.48/0.43 [0.05]; mean absolute edge weight 0.27/0.27 [0.04]; positive edges 99.7/97.0% [56%]; Fig. 4*A* and *SI Appendix*, Fig. S6). A similar loss of structure was observed for the untreated network projections (wild fall/spring [laboratory]: density = 0.39/0.32 [0.07]; mean absolute edge weight 0.23/0.20 [0.05]; positive edges 93.3/93.0% [60%]). Differently from the abiotic and untreated networks, the topology of the biotic network was strongly preserved across both seasons in the wild (wild fall/spring [laboratory]: density 0.33/0.32 [0.27]; mean absolute edge weight 0.21/0.19 [0.16]; positive edges 99.3/99.3% [86%]). While the stronger preservation could reflect that the more highly connected topology of the biotic network transfers more robustly across conditions, the pattern is consistent with downstream responses to diverse biotic challenges being more similar than responses to diverse abiotic stressors.

How much the observed network topology shifts was due to changes within modules versus relationships between modules? To answer this question, we used module preservation scores (36) to separate modules into highly conserved between the laboratory and the wild (*Z*-summary > 10), moderately conserved (2 < *Z*-summary ≤ 10), and unconserved (*Z*-summary ≤ 2). Distribution of modules across these three classes differed significantly among networks (χ^2^ = 12.79, *P* = 0.012, *SI Appendix*, Fig. S9 and Dataset S12). Specifically, the biotic modules had about 70% lower odds to be moderately preserved than the untreated and abiotic network modules (Fisher’s exact *P* = 0.002). Conservation also varies by function: cell cycle modules are among the most conserved, while secondary metabolism modules are among the least conserved.

To examine changes in between-module topology, we constructed hub-gene networks. Hub genes were defined as the top 10% of genes by within-module connectivity (kIN) in each module (Datasets S13 and S14). In the laboratory biotic network (Fig. 4*B*), between-module links were concentrated among cell-cycle modules, between cell-cycle and transcription-related modules, and between photosynthesis and transcription-related modules, whereas stress-response and secondary-metabolism modules remained relatively isolated. In the wild-projected biotic hub network, interconnections among cell-cycle modules persisted, but overall between-module connectivity increased markedly—most notably for hubs from the red (secondary metabolism), white (translation), and cyan (stress response) modules (Fig. 4 *B* and *C*, *SI Appendix*, Fig. S10, and Dataset S14).

A key shift was the largest module (pink; *N* = 5,881; GO: transcription) becoming a major intermodule hub, gaining strong new links to multiple stress-response modules (Fig. 4*C*). This pattern suggests tighter coupling between transcriptional regulation and stress or defense responses, and thereby likely strengthening coregulation between defense- and growth-related functions. Notably, modules with high levels of intermodule hub gene connectivity in both the laboratory and wild are the ones with high conservation scores (Fig. 4*D*). Abiotic hub-gene networks (*SI Appendix*, Fig. S10) largely mirrored the ME networks (Fig. 4*A*): The abiotic hub network from the laboratory had sparse intermodule connectivity, whereas the wild-projected hub network had many intermodule connections, with little evidence of well-defined module boundaries or preferential connection between modules with related GO terms.

We also performed RDA on the wild-projected modules. Microbial infection explained more variation for modules associated with immunity or stress responses (e.g., yellow-green module [systemic acquired resistance], light-green [response to SA], cyan [response to hypoxia]; *SI Appendix*, Fig. S11). Taken together, these results close the loop between biotic network modules derived from well-defined, single treatments in the laboratory and our field observations. The relative lack of module associations with abiotic stimuli might reflect the limited range of environments we sampled compared to the species’ native range, the severity of abiotic stimuli used in typical laboratory experiments, or failure to fully capture microenvironmental variation in abiotic factors between plants within a site and during their life history.

### Prediction of Bacterial Infection with Module Eigengenes.

**Fig. 5.**
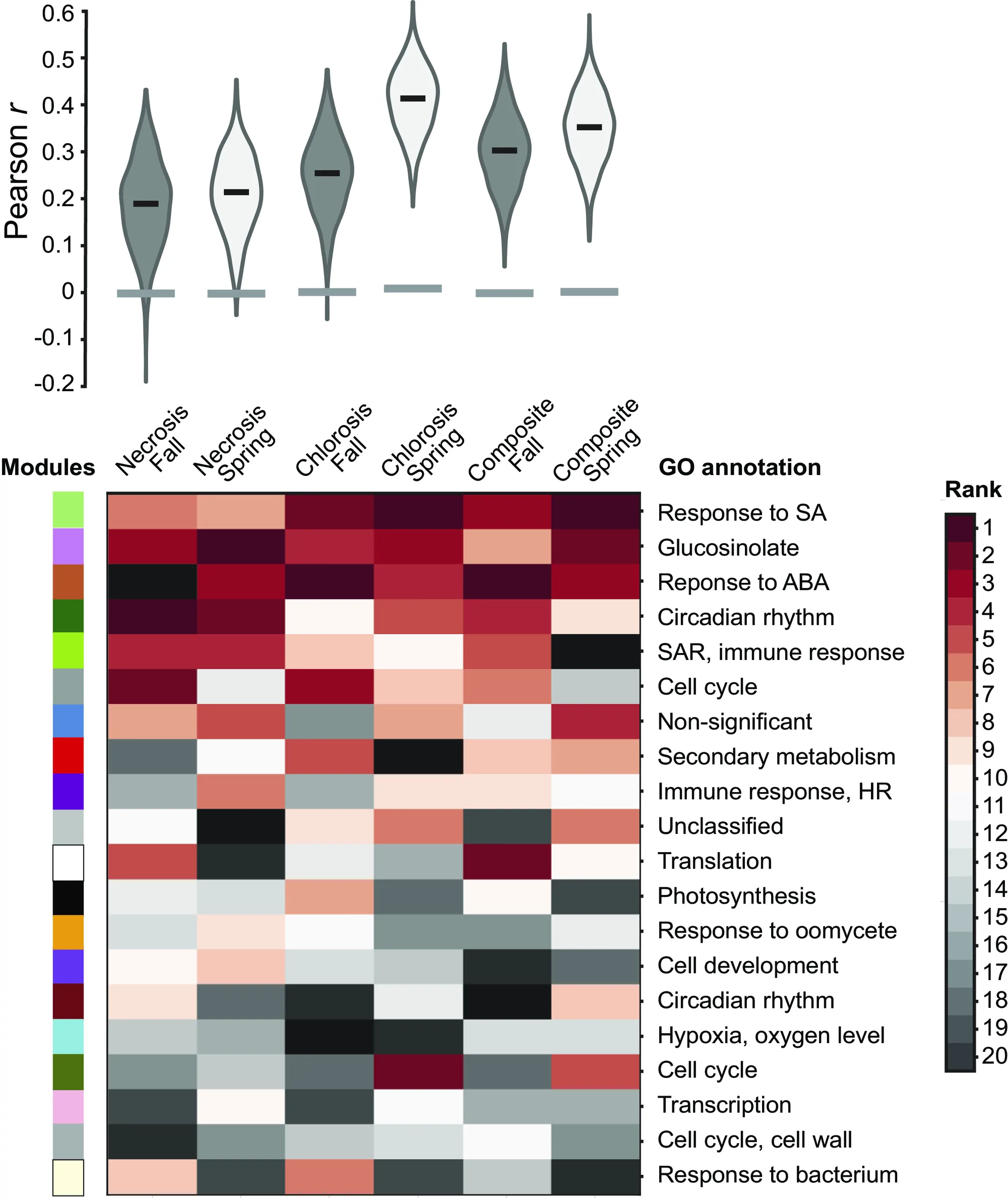
Prediction of bacterial-infection related field traits with projected MEs. Random forest prediction of necrosis, chlorosis, and their composite score using MEs derived from wild-projected modules in the biotic WGCNA network. (*Top*) Pearson’s *r* between the predicted and true value. Violins illustrate the distribution of *r* in 1,000× random forest iterations. Dark gray, fall; light gray, spring. Horizontal gray bar at the bottom of each violin indicates the median value of 1,000× permuted null distributions. (*Bottom*) Heatmap illustrating ranks of feature importance scores for each ME in the six predicted traits-season combinations. Modules are arranged from top to bottom based on their median ranks for the six combinations. For module annotation by color, see Dataset S11.

To further investigate the association of the wild transcriptomes with phenotypes observed in the field, we adopted a random forest approach. We used module eigengenes (MEs) from the projected biotic modules to predict infection-related field scores, focusing on necrosis and chlorosis because of their prevalence and quantitative nature across sites and seasons. We assessed significance by repeating Monte Carlo cross-validation with 1,000 permutations of phenotype labels. Correlations with MEs were consistently higher than expected by chance (Fig. 5). Predictive performance of MEs was comparable between seasons for necrosis (median/interquartile range of Pearson’s correlation coefficients: fall 0.19/0.13, spring 0.21/0.11) and the necrosis/chlorosis composite score (fall 0.30/0.11, spring 0.35/0.10). Chlorosis alone was more predictable than necrosis in both seasons, especially in spring (fall 0.25/0.11, spring 0.41/0.10). This suggests that chlorosis in spring might be a more integrative phenotype, for example, because it is the result of multiple disease pressures, increased vulnerability due to onset of leaf senescence, or modified defense allocation upon bolting (*SI Appendix*, Fig. S8). In fall, the combination of necrosis and chlorosis increased predictability, suggesting partially distinct contributions of these components across seasons.

Across traits and seasons, most MEs (16 to 20 of 20) performed significantly better than the null expectation (*SI Appendix*, Fig. S12). Five modules repeatedly ranked among the top predictors (Fig. 5). They were enriched for SA response (median rank 2.5/significant traits 6), ABA response (2.5/5), glucosinolate biosynthesis (3/6), systemic acquired resistance/immune response (4.5/6), and circadian rhythm (5/6). The stable correlation between module expression levels and field scores could be a consequence of hardwired transcriptional plasticity and/or reflect the activity of a small set of polymorphic upstream regulators. An example of the latter is the strong effect of common genetic variants on the coregulation of many downstream genes in the aliphatic glucosinolate biosynthetic pathway (39, 40). These findings corroborate that transcriptome variation is physiologically relevant, with infection-related modules explaining a reproducible fraction of the observed disease-score variance. Modules enriched for cell cycle and translation also frequently contributed to predictions, in agreement with coordination between growth-associated processes and defense in the wild.

### High Variation in Expression of General Non-Self Response Genes.

**Fig. 6.**
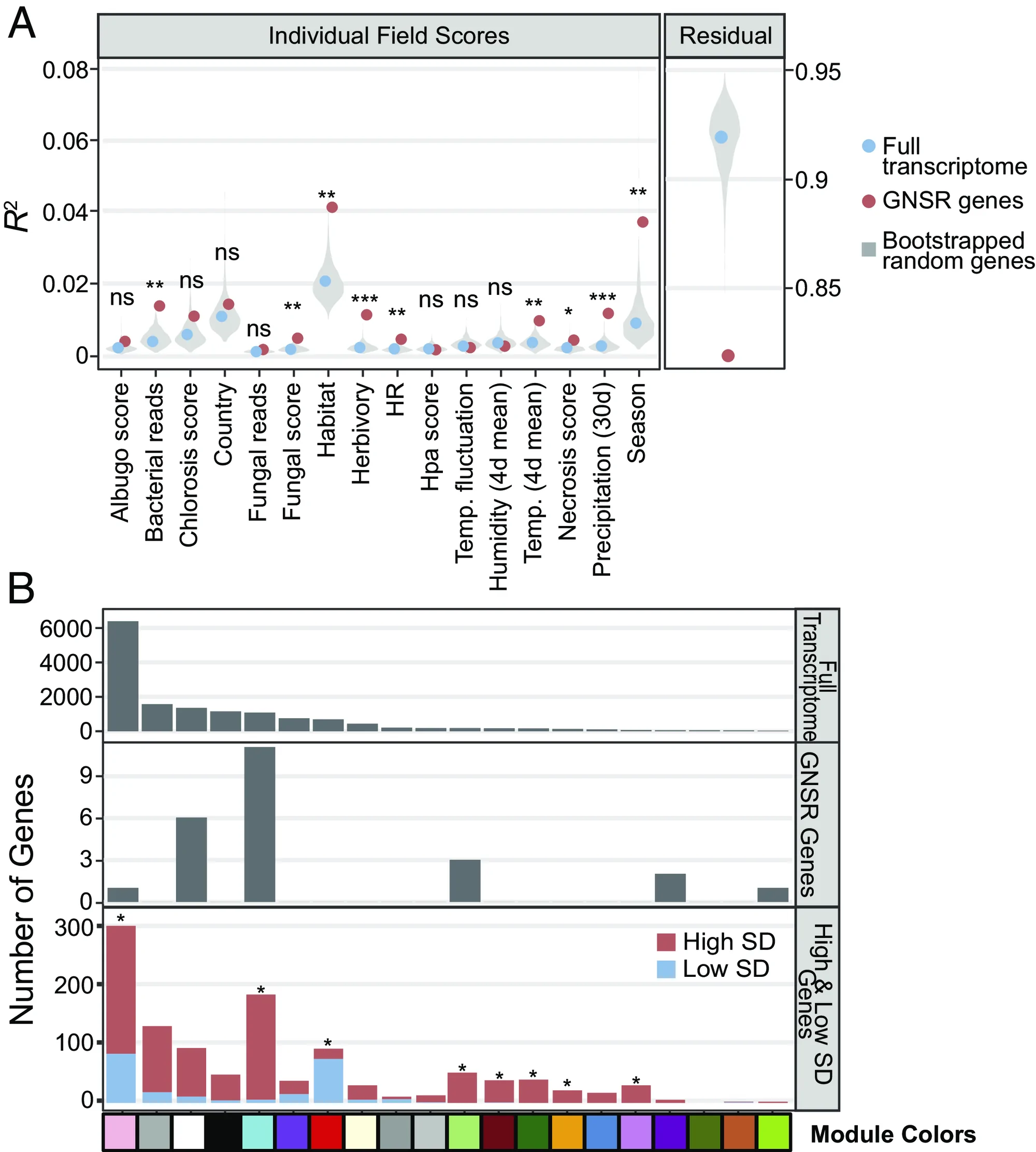
Association of general non-self response (GNSR) gene expression with field scores. (*A*) Variance explained by various field scores, as inferred by PERMANOVA. Each variable was used to model variance in the full transcriptome (blue dots), among the 24 GNSR genes (red dots) and 24 random genes (1,000× bootstraps, gray violins). Asterisks indicate significance when comparing 24 GNSR genes and 24 random genes. ***P < 0.0005; **P < 0.005; *P < 0.05; ns, nonsignificant. Residual variance after PERMANOVA is also lower for GNSR genes in comparison to both the full transcriptome and 24 random genes. (*B*) WGCNA module membership of GNSR genes (*Middle*) and low- and high-SD genes (*Bottom*) is not explained by module size (*Top*). Asterisks indicate significant enrichment of high-SD genes in a module (Fisher’s exact *P* < 0.005). Note that most of the low-SD genes (710 of 922) were not found in WGCNA modules, in agreement with limited expression variation. For module annotation by color, see Dataset S11.

Because it is difficult to know which specific microbes are most relevant for wild plants, we analyzed the expression of genes that are activated in response to many different bacteria. This set of 24 general non-self response (GNSR) genes, which was originally identified based on activation by diverse bacterial taxa, captures a broad transcriptional response to non-self microbial cues (41). We compared the proportion of expression variance explained by latent estimates of plant state between the whole transcriptome and GNSR genes using PERMANOVA and tested significance by comparing the variance explained by 24 GNSR genes to that of 24 random genes. Latent estimates explained considerably more variance in GNSR genes, 19%, than in the global transcriptome, 9%, or in 24 random genes, 5 to 15% (1,000 bootstraps, comparison to 24 GNSR genes *P* = 0.001, Fig. 6*A*). Individually, bacterial and fungal infection, herbivory, habitat, mean temperature, total precipitation, and season had significantly higher explanatory power for GNSR genes than for 24 random genes (Fig. 6*A*). These results are in agreement with GNSR genes being highly responsive to many diverse pathogenic bacteria.

GNSR genes were predominantly assigned to the previously defined biotic WGCNA modules with annotated roles in responses to stimuli (4/8 modules) and transcription/translation, and modules whose activity correlated with bacterial infection (Fig. 6*B* and *SI Appendix*, Fig. S11). Moreover, 13 of the 24 GNSR genes were high-SD genes (Fig. 6*B*). High-SD genes as a group were similarly overrepresented in modules with annotated roles in responses to stimuli (5/8) and transcription/translation (Fisher’s exact *P* < 0.005, Fig. 6*B*), which supports the idea that the connections between these two groups of processes are strengthened in the wild. Together, these results suggest that responses to the multitude of challenges faced by each plant drive highly variable expression of key genes, and that genes with highly variable expression, far from being simply noisy, may prove an important resource for understanding the transcriptional states of wild plants.

## Discussion

We have asked how much the organization of transcriptomes into functional modules aligns between wild individuals and plants that have been grown under defined laboratory conditions. By sampling across multiple regions and seasons and combining host transcriptomes with environmental, microbial, and phenotypic data, we found extensive transcriptome variation that cannot be easily attributed to single major factors such as geography, season, growth stage, or obvious biotic or abiotic stress phenotypes. Transcriptomes were, however, not randomly organized. Using gene coexpression networks derived from laboratory datasets as a guide, we found that wild transcriptomes most closely resembled networks revealed in response to biotic challenges in the laboratory, whereas networks derived from control conditions or abiotic stresses were substantially reorganized in the wild. The absence of discrete clusters of distinct global transcriptomes in the wild suggests that the degree to which these networks are deployed is largely continuous, in stark contrast to the discrete activation of different transcriptional programs observed in response to individual stressors in the laboratory.

Measured environmental and phenotypic factors explained only a small fraction of total transcriptomic variance, most likely reflecting the complexity of gene expression states in natural populations. Nevertheless, within this constrained component of variance, latent variables representing microbial infection explained more variation than developmental stage or recent weather summaries. We do not think that this implies a lesser importance of abiotic factors in nature, but rather that, under the range of environments sampled here and given the variables measured, infection-related signals leave a more detectable imprint on transcriptome variation. One potential explanation is that sharing of downstream signaling pathways is more common for biotic than abiotic stresses (42). That disease field scores on their own explained only a small fraction of transcriptome variance could have many different reasons, such as asymptomatic infections (43), lasting effects of early infections (44), or the relationship between visual symptoms and molecular responses changing with plant age (45). Additionally, in any observational study such as ours, statistical variation is more readily attributed to explanatory factors that are measured more precisely and at a finer individual level (in our case, primarily biotic interactions). We aimed to minimize this bias through latent estimation of microbial infection, but there are always many more unobserved forces influencing the physiological state of a wild plant, as evidenced by the large proportion of unexplained variance.

Network comparisons provided additional insight into how transcriptional regulation apparently differs between laboratory and natural contexts. Modules representing core biological processes, such as transcription, metabolism, growth-related functions, and stress responses, were recognizable in our data, supporting the view that functional organization of the transcriptome is broadly conserved between the laboratory and the wild. However, relationships among modules differed substantially. In particular, abiotic-response and untreated networks from the laboratory lose much of their modular structure in the wild, while biotic-response networks retain much of their topology. Thus, transcriptional programs characterized under biotic challenges in the laboratory map more coherently onto expression states observed in wild populations than those derived from abiotic perturbations or untreated controls.

A notable feature of wild transcriptomes is the increased connectivity between modules associated with stress and defense responses and those linked to transcriptional and translational regulation. Hub gene analyses showed that modules related to transcription and translation occupy more central positions in projected wild networks, forming connections with both stress-response and growth-associated modules. These patterns suggest that, under natural conditions, coexpression programs associated with growth and defense are more tightly coordinated than what is observed in the laboratory, where responses are often measured after acute stimuli and in isolation. Testing this possibility directly will require integrating network analyses with explicit measurements of growth and fitness in the wild.

The relationship between growth and defense responses has long been discussed in terms of trade-offs, often, but not exclusively, based on laboratory observations where constitutive immune activation generally leads to growth penalties (46–49). Direct measurements of defense costs in natural populations or even crops have, however, yielded mixed results (50–55). Our results offer an alternative perspective, that differences in network connectivity between laboratory and wild conditions contribute to differences in observed growth–defense relationships across environments. At the same time, our wild sampling necessarily included only plants that survived until collection, leading to underrepresentation of individuals that grew very poorly or that succumbed to acute pathogen attack. Such survivorship bias could weaken the manifestation of a growth–defense trade-off in the wild, especially because laboratory experiments often use few genotypes and strong, synchronized perturbations. However, previous field and common-garden studies in native *A. thaliana* environments suggested that postgermination mortality is often modest and can be driven not only by pathogen pressure but also by abiotic microhabitat conditions (9, 56). We therefore interpret the reduced coupling between growth- and defense-associated modules in wild plants not as evidence of the absence of genetic trade-offs, but as the combined outcome of ecological filtering, genotype-by-environment interactions, and heterogeneous stress response in nature. Another striking observation is the large fraction of transcriptome variance that remains unexplained by measured variables. This likely reflects both biological and technical factors. Sampling from the wild captures heterogeneous developmental histories and microenvironmental exposures that are difficult to measure comprehensively.

Analyses of genes previously identified as part of the general non-self response (GNSR) (41) further support the view that immune-related pathways are dynamically regulated in natural populations. Variation in expression of GNSR genes is particularly high, their expression is strongly associated with measured field variables, and several belong to WGCNA modules related to stimulus responses and transcriptional regulation. While we do not know whether this variability is indeed adaptive, it indicates that genes broadly responsive to microbial signals remain highly dynamic in wild populations and contribute substantially to observed transcriptional heterogeneity.

Our study has a series of limitations that could be addressed in future work. Different potential drivers of the wild transcriptome could not all be measured with equal resolution: infection-related variables were assessed at the individual-plant level through symptom scoring and metagenomic profiling, whereas abiotic variables were summarized from nearby weather stations and may not capture plant-scale microenvironmental heterogeneity. We do not know how exactly plants integrate environmental exposures over longer timescales, and differences in soil properties may also contribute to variation that cannot easily be partitioned using available metadata. Thus, our conclusions should be read as infection-associated signals being strongest among the variables measured here, not as evidence that unmeasured abiotic or biotic factors are unimportant. More generally, temporal, developmental, and microenvironmental heterogeneity likely reduces power to attribute transcriptome variation to individual drivers, rather than generating coherent preservation of laboratory-derived response networks.

Disentangling the multitude of potential drivers of transcriptome variation in natural populations may require reducing heterogeneity by focusing on individuals based on synchronized germination and shared life history or measuring environmental variation at finer spatial and temporal resolution. Dense longitudinal sampling of plants across developmental stages could further clarify how transcriptome states integrate environmental history. An informative contribution in this regard comes from a recent investigation of morphological phenotypes and transcriptomes of native *A. thaliana* plants (19), a study with complementary insights to our work, based on sampling hundreds of individuals per site and year at two native sites in Germany across 5 y. By focusing on leaf-surface temperature measurements, Mjema and colleagues (19) identified the expression of known key regulators of thermomorphogenesis as predictors of temperature-associated transcriptome variation. In comparison, our seasonal sampling was designed to minimize developmental contrasts between sampling windows, while the broader geographic sampling came at the cost of the number of samples per site. Our results show that, once the major axis of developmental variation is held relatively constant, the dominant organizer of transcriptome variation is biotic in nature, with infection-associated signals accounting for more of the explainable variance than recent weather summaries. We conclude that temperature and biotic stress are not competing explanations for the organization of wild transcriptomes but rather operate at different temporal and spatial scales. Temperature impacts broad seasonal and year-to-year trajectories detectable when the same sites are sampled at depth, whereas pathogen-associated signals emerge as the primary structuring force when transcriptomes are sampled across the full ecological heterogeneity of the species’ range. Finally, while most of the variation in native transcriptomes remains insufficiently explained by variables measured in the field in both studies, together they begin to provide a path forward for designing sampling strategies across space and time that capture more of the factors that shape native transcriptomes.

At a broader level, our results support a systems-level perspective in which functional gene modules identified in laboratory experiments remain relevant in natural populations, but their organization and activity differ across environments. Laboratory experiments excel at identifying modules and causal pathways under defined perturbations, whereas natural environments reveal how these modules are coordinated under sustained and heterogeneous conditions. Integrating both approaches offers a path toward understanding how molecular regulation operates in realistic ecological contexts and how coexpression networks are deployed across environments.

## Materials and Methods

We sampled whole rosettes of plants from 60 sites across southern France, southwestern Germany, and the midwestern United States in fall 2022 and spring 2023, targeting 12 plants per site and season. For each individual, we recorded developmental traits, symptoms of bacterial, fungal, and oomycete infection and herbivory. Recent local weather data were from nearby public weather stations. DNA and RNA extracted from each rosette were used for whole-genome shotgun and RNA sequencing. Reads were mapped to the *A. thaliana* reference genome for variant calling and additionally classified taxonomically with a custom Kraken2 database to estimate microbial abundance. Field observations and sequencing-based infection measures were integrated using confirmatory factor analysis to derive latent estimates of bacterial, *Hyaloperonospora* and *Albugo* infection. Rosette diameter and leaf number were combined into a latent estimate of developmental state. After quality control, the RNA-seq dataset included 616 samples and 18,452 expressed genes. Transcriptome-wide variation was first assessed by PERMANOVA and PCA, followed by correction for library-plate effects while retaining variation associated with sampling time, country, and season. We constructed a de novo WGCN from the field transcriptomes using WGCNA, summarized each module by its eigengene, and related module activity to infection, developmental state, and weather. Redundancy analysis was used to partition module-level transcriptomic variation among these explanatory variables. To place observed coexpression patterns in the context of controlled perturbations, we curated 2,849 public RNA-seq samples and generated separate WGCNA networks for untreated, abiotic-stress and biotic-stress conditions. Laboratory-derived modules were projected onto the wild transcriptomes, and their preservation, eigengene correlations, and higher-order network structure were compared across laboratory and field conditions. Module-eigengene networks were analyzed for connectivity, density, edge strength, sign structure, and functional assortativity. Hub genes, identified from within-module connectivity, were used to construct reduced hub-gene interaction networks. Random-forest models with repeated train/test splitting were used to test how well module eigengenes predicted field phenotypes and metagenomic traits and to estimate the contribution of individual modules to these predictions. Full methods are provided in the *SI Appendix*.

## Supplementary Material

Appendix 01 (PDF)

Dataset S01 (XLSX)

Dataset S02 (XLSX)

Dataset S03 (XLSX)

Dataset S04 (XLSX)

Dataset S05 (XLSX)

Dataset S06 (XLSX)

Dataset S07 (XLSX)

Dataset S08 (XLSX)

Dataset S09 (XLSX)

Dataset S10 (XLSX)

Dataset S11 (XLSX)

Dataset S12 (XLSX)

Dataset S13 (XLSX)

Dataset S14 (XLSX)

Dataset S15 (DOCX)

## Data Availability

Analysis code is available at https://github.com/kdm9/montalvao-rnaseq/ (57). Sequencing reads are available at the ENA under the project number PRJEB109258 (https://www.ebi.ac.uk/ena/browser/view/PRJEB109258) (58). Additional data files are available at figshare at https://doi.org/10.6084/m9.figshare.31638136 (59). In addition to Datasets S1–S14 as “*SI Appendix*, Tables S3.1–3.14,” these include the metagenomic profiles of wild plants (two files), the normalized read counts of wild RNA-seq samples, and the normalized read counts of public RNA-seq samples. All other data are included in the article and/or supporting information.
