# Supplementary material for: Biotic response networks are an important organizer of the transcriptome in wild Arabidopsis thaliana populations
Source: Proc Natl Acad Sci U S A. 2026 Oct 19;123(43):e2611539123. doi: 10.1073/pnas.2611539123 (PMC13715808; doi:10.1073/pnas.2611539123)
Supplement: Supplementary file 1 [file pnas.2611539123.sapp.pdf]

PNAS

Supporting Information for
Biotic response networks are an important organizer of the transcriptome in wild *Arabidopsis thaliana* populations

Ana Paula Leite Montalvão*, Kevin D. Murray*, Ilja Bezrukov*, Natalie Betz, Lucas P. Henry, Paloma Duran, Patrick Boppert, Martina Kolb, TEAM PATHOCOM[#], Fabrice Roux, Joy Bergelson, Detlef Weigel, Wei Yuan

[#]Haim Ashkenazy, Tien-Hao Chang, Choghag Demijirian, Rémi Duflos, Alejandra Duque-Jaramillo, Katrin Fritschi, Andrew Gloss, Anette Habring, Katharina Klein, Eric Laderman, Irene Leccia, Roxane Lion, Hanna Märkle, Baptiste Mayjonade, Timothy C. Morton, Ankita Pandey, Alexandre Perrat, Kamal Radharamanan, Angelique Rat, Sonia Silva, Manmeet Singh, Daniel de Souza, Hannah Whitehurst

*Equal contribution

Corresponding authors: Detlef Weigel, Wei Yuan
Email: weigel@tue.mpg.de (D.W.), wyuan8832@gmail.com (W.Y.)

This PDF file includes:

Extended Methods
Figures S1 to S12
SI References

Extended Methods

Wild plant sampling

Full rosettes were collected from 60 wild sites of *Arabidopsis thaliana* across southern France, midwestern USA, and southwest Germany. Sampling was conducted during fall 2022 (November-December) and spring 2023 (February-April), with exact timing depending on the local conditions in each country (Fig. 1). At each site and season, we aimed to collect 12 individual plants within two to three weeks. For each plant we recorded signs of herbivory (0-5), hypersensitive response-like symptoms (P/A), as well as symptoms of bacterial disease (chlorosis, necrosis; 0-5), which were used both individually and as a composite score of their sum, and symptoms of infection by fungi, *Hyaloperonospora* spp., and *Albugo* spp. (0-5). (Fig. 2A, Fig. S2, Dataset S1). The developmental state was characterized by counting the number of leaves, rosette diameter, and the presence or absence of bolting. We extracted weather data from nearby weather stations (Dataset S2) from wunderground.com for the duration of the sampling season (October 1, 2022, to April 30, 2023). For each sampling visit, we summarized the weather data as mean temperature of the four days preceding sampling, mean humidity for the four days before sampling, mean diurnal temperature range for the four days before sampling, and sum of precipitation during the 30 days preceding sampling. We summarized these variables through the first two principal components (PCs) in a principal component analysis (PCA).

RNA and DNA extraction

DNA and RNA were simultaneously extracted from individual rosettes. After scoring, wild-collected rosettes were washed with sterile distilled water to remove surface soil, then placed in 2-ml screw-cap tubes containing a mix of glass and ceramic beads (equal volumes of sterile 0.2mm, 0.5mm, 1.5mm ceramic beads, and one 5mm glass bead, dispensed with a Custom Lab Institute 96 well Powder Dispenser), frozen on-site in dry ice and stored at −80°C until extraction. Samples were homogenized to a fine powder using a Precellys Tissue Homogenizer (Bertin Technologies, Montigny-le-Bretonneux, France) equipped with a Cryolys cooling adapter, using two cycles of 15 s at 6,300 rpm. Nucleic acids were purified using ECONOSPIN columns (Epoch Life Science, Inc., Fort Bend, TX, USA) adapted to 96-well plates following the protocol described in (1). Briefly, 800 µl of extraction buffer (20 mM EDTA, 8M guanidine HCl, 20 mM MES) was added to each sample. Homogenized samples were mixed with equal volume of absolute ethanol before loading the mixture onto column plates. Samples were sequentially washed 3M sodium acetate, twice with 70% ethanol before elution in 60 µL nuclease free water. Aliquots of eluates were subjected to RNase A or DNaseI (Thermo Fisher) treatments to retrieve DNA and RNA fractions, respectively. The products were stored at −80 °C.

Library preparation and sequencing

We constructed whole-genome shotgun DNA sequencing libraries using an in-house tagmentation protocol similar to Nextera Flex, originally derived from (2). Briefly, we normalized DNA to a maximum concentration of 5 ng/µL, tagmented the DNA with bead-bound Tn5 transposase, then amplified with dual-index primer pairs with 12 cycles of PCR using Q5 polymerase (NEB). Cleaned up libraries were pooled at equal volume across a plate. Plate pools were subjected to size selection (300-750 bp) using BluePippin (Agilent Technologies, Santa Clara, CA, USA), with final fragment size assayed on the Bioanalyzer platform (Agilent Technologies) and pooled across plates equimolarly. We sequenced libraries to a mean yield of 6 Gb on an Illumina Novaseq X Plus 25B instrument with 2x150bp paired end reads.

We constructed RNA-seq libraries based on an in-house protocol (3). Briefly, we used ~1 µg of total RNA from full rosettes, synthesized cDNA, selected mRNA with oligo-dT bead-pulldown, then produced libraries with unique dual-indexed barcodes. We pooled libraries at equimolar ratios and sequenced the pool to a mean yield of 2 Gb per library.

Metagenome analysis

We used shotgun DNA sequencing reads to determine the ratio of microbial and plant genomes, to call plant genotypes, and to assign microbial reads to different taxa. We analyzed WGS reads with the Acanthophis pipeline (4). Briefly, we performed read QC with AdapterRemoval (5), trimming adapters and low-quality sequences from reads, and removing reads that were shorter than 64bp after trimming. We mapped reads to the *A. thaliana* TAIR10 reference genome (6) with BWA MEM (7), and called variants with DeepVariant and GLNexus, using the DeepVariant-WGS model (8, 9). We filtered variants as follows: samples were removed if they had less than 10 total read depth (aggregated over SNP calls), or had more than 5% missing genotype calls, and sites were removed if they had more than 5% missing data, or fewer than 3 observations of the less common allele.

To allow deeper taxonomic profiles without excessive RAM use, we used a hierarchical reference set to create a custom kraken2 database. Specifically, we took all RefSeq representative bacterial genomes, and all RefSeq representative genomes from Brassicaceae. To this, we added all complete *Arabidopsis* spp. genomes from the NCBI genomes portal, and additional 77 *A. thaliana* long read genomes (10, 11), as well all complete genomes for every bacterial genus in At-LSPHERE (12). We used kraken2 (13) to assign all reads passing QC to this custom *A. thaliana*-specific reference database, and to the Kraken2 PlusPFP reference database. We used the proportion of basepairs assigned to each microbial family divided by either the total library yield (prop_taxon) or the number of basepairs assigned to Brassicaceae (taxon_per_plant) respectively, using R and DuckDB. We also summarized to higher taxonomic levels: Oomycota, Fungi, and Bacteria, reported as both proportions and host ratios as for family-level summaries above, along with Other, representing reads that were positively classified but not to one of these higher level groups, and Unclassified reads that could not be assigned to any taxon (https://doi.org/10.6084/m9.figshare.31638136).

Latent variable estimates of infection

To better integrate the multiple unreliable indicators of microbial infection (including visual scores and taxonomically-ambiguous microbial reads), we used confirmatory factor analysis to predict latent variables that represent the consistent signal of these indicators. To do so, we modeled infection as a function of all observed variables likely to be correlated with a genuine infection using cfa from the R package lavaan (14). We modeled infections of bacteria (infection ~ necrosis + chlorosis + hr + prop_pseudo + prop_xantho + prop_sphingo + bact_family_entropy), *Albugo* spp. (infection =~ albugo + prop_albugo), and *Hyaloperonospora* spp. (infection =~ hyalo + prop_hyalo), producing predictions of infection that incorporate variation across both field observations and sequencing data. We also fit a model of developmental state, as (dev_state =~ rosette_diam_mm + num_leaves). Bolting was not included in the developmental state estimates as it is a binary trait largely nested within season leading to unstable model fit. Overall, we found the model fit to be good: cfi=0.896; tli=0.848; rmsea=0.063; srmr=0.047 (Fig. S3).

RNA-seq analysis

We first quality controlled short RNA-seq reads with AdapterRemoval (5), then down-sampled 132 high-coverage samples to no more than 10 million reads using seqtk (15). We then mapped RNA-seq reads to the *A. thaliana* TAIR10 reference genome with Araport11 annotation (16), and estimated expression of individual gene models with RSEM (17). A Snakemake pipeline for these steps is available at https://github.com/kdm9/montalvao-rnaseq (18). We filtered RNA count tables as follows: we removed organellar transcripts, transcripts mapped to rDNA, rRNA, nucleolar genes, pseudogenes, pre-tRNAs, and transposable elements, as well as transcripts with RSEM-estimated effective length less than 150 bp, whose count estimate tends to be inaccurate (19). This left 30,669 genes for further consideration.

We then removed samples with fewer than 2 million mapped reads, leaving a total of 616 usable samples. We applied the Variance-stabilizing transformation (VST) on the filtered raw-counts of these samples using DESeq2 command vst (20). We further filtered the normalized count matrix to

remove genes with zero expression in more than 580 (95%) samples, extremely low average expression (vst ≤ 4.2), and either extremely low (coefficient of variation ≤ 0.01) or extremely high variance (coefficient of variation ≥ 0.23). We determined all thresholds manually after examination of the histograms of the respective parameters. We then created the normalized expression matrix of 18,452 genes and 616 samples (https://doi.org/10.6084/m9.figshare.31638136).

A first look at transcriptome variance (PERMANOVA (21)) identified sampling time (hours after sunrise), country, and season to be the major source of systematic biological variation, while plate effects (i.e., on which 96-well plate a library had been synthesized) drives major batch noise. To correct for batch effect while preserving biologically meaningful signal, we used Limma's removeBatchEffect (22, 23), while specifying effects to be kept using optional argument design= model.matrix(~ hours_after_sunrise + country + season). PCA was run before and after to evaluate the efficiency of batch effect removal.

Analysis of high- and low-SD genes

We calculated the standard deviation across samples for each gene using variance stabilized expression values. The 5% of genes with the highest and lowest SD were considered high- and low-SD genes (N=922 genes for each group). GO term enrichment was performed using PANTHER (24) with FDR-corrected *p*-values. To determine whether high- and low-SD genes were similar across countries and seasons, we subset samples (e.g., France + Spring), and re-calculated standard deviation.

De novo WGCNA generation

All expressed genes after initial filtering (*N*=18,452) were used in WGCNA to minimize sensitivity to arbitrary preprocessing choices and to avoid selection bias introduced by post hoc filtering or subsetting. A signed WGCN (25) were generated using biweight midcorrelation (bicor; maxPOutliers = 0; maxBlockSize=10,000). Soft-thresholding powers from 1-30 were evaluated using the scale-free topology criterion, and a power of 6 was selected as the lowest value achieving signed $R^2 \geq 0.8$. Modules were identified using blockwiseModules (minimum module size = 30, deepSplit = 4, pamRespectsDendro = FALSE), and similar modules were merged at a cut height of 0.15. MEs were derived as values of the first PC of each module. Associations between MEs and external variables were assessed using Pearson's correlation.

Modeling transcriptome variation using latent estimates

We used Redundancy Analysis (RDA) to model variation among genes in each of the 18 wild WGCNA modules as a function of latent estimates of infection, development, and summaries of weather data. For each module, we fit an RDA across all genes in the module (removing between 0 and 2 genes per module with perfectly correlated gene expression to avoid ill-defined models). We used latent estimates of infection with bacteria, *Hyaloperonospora* spp., and *Albugo* spp. as well as developmental stage, and recent weather PC1 and PC2 as constraining factors in each RDA model. We summarized the variance in each model hierarchically as follows: proportion of total transcriptome variance contained in this module, proportion of module variance constrained by the explanatory factors, and proportion of constrained variance attributable uniquely to each constraining factor.

Curating public RNA-seq data

We downloaded FPKM values for an ensemble of 28,164 *A. thaliana* RNA-seq samples and their corresponding metadata from https://plantrnadb.com (26). Almost all samples were from the Col-0 reference strain (Dataset S8). We included experiments with more than five samples (i) when treatments were clearly annotated as biotic or abiotic, (ii) when treatment and control samples could be distinguished, and (iii) when genotype and tissue metadata were unambiguous. Biotic treatments included pathogen, microbe, herbivore, and defense-hormone experiments, whereas abiotic treatments included non-living environmental perturbations such as temperature, water availability, salt, light, and related physical or chemical stresses. We excluded samples with

combinatorial treatments to enable direct comparison of co-expression structures learned from single-stressor laboratory experiments with wild transcriptomes, where plants experience multiple overlapping cues. We manually curated metadata and coded a binary treatment-by-sample design matrix across 24 biotic and abiotic treatment categories for downstream WGCNA module-trait associations, yielding 1,118 control/mock samples, 724 abiotic treatment samples, and 1,007 biotic treatment samples (Dataset S8; https://doi.org/10.6084/m9.figshare.31638136).

Comparative meta-WGCNA

Curating public gene expression data: We removed genes with >50% dropout rate, mitochondrial, chloroplast, rDNA, rRNA, and nucleolar genes, pseudogenes, pre-tRNAs, transposable elements, and genes shorter than 150 bp. Genes with extremely low average expression, and globally invariant genes were filtered separately for the abiotic/biotic treatment datasets and the untreated samples based on quantile distribution (abiotic/biotic: mean log(FPKM) ≤1, SD ≤ 1.15; untreated: mean log(FPKM) ≤1, SD ≤ 0.99). This left 14,854 genes for the abiotic/biotic samples, and 13,630 genes for the untreated samples.

WGCNA network construction: We generated signed WGCNA networks (25) biweight midcorrelation. Soft thresholding power (Untreated=13, Abio=12, Bio=20) was evaluated with the pickSoftThreshold function (signed, bicor) on a series of thresholds from 1-30, and determined using scale-free fit ($R^2 \geq 0.8$) and mean connectivity (Untreated:51.39, Abio: 63.99, Bio: 24.92). Deep split values (Untreated: 3, Abio: 4, Bio: 2) were chosen by running 100x boot straps of 80% of the samples on allowed deepSplit values (2-4) for each dataset and evaluating the module robustness using adjusted Rand Index (ARI) and Jaccard Index. Similarly, optimal tree-cut height (Untreated: 0.15, Abio: 0.2, Bio: 0.1) was determined by running a series of cut heights and bootstrapping the merged modules for ARI and Jaccard index as stability measurements.

We constructed gene co-expression networks with WGCNA step-wise using functions adjacency (signed, bicor), TOMsimilarity, cutreeDynamic (minimum module size=30), and mergeCloseModules. After each gene had been assigned to a module, we calculated MEs as the first PC for all genes in a module, using the WGCNA function moduleEigengenes. GO biological process enrichment was performed for each module using packages clusterProfiler (*v4.10.1*, (27)) and org.At.tair.db (*v. 4.5*, (28)). GO enrichment results were manually curated to determine a single representative GO annotation for each module (Dataset S11). We calculated Pearson's correlation coefficients to establish associations between MEs and treatments.

Network projection: For each season, genes in the wild RNA-seq data set were assigned to modules in the untreated, abiotic, and biotic networks. Expression of most genes was detected in both laboratory and wild data: of 14,854 genes included in the laboratory abiotic/biotic networks, 13,256 were also in the wild data, and for the untreated network, of 13,630 genes, 12,093 were also in the wild data. ME values in the projected networks were calculated using the WGCNA function moduleEigengenes. We calculated Pearson's correlation coefficients to establish associations between MEs and the field scores.

Constructing ME networks: We calculated the ME adjacency matrices by arbitrarily raising the ME correlation matrices to the power of 4. A range of powers was tested to make sure that results with a power of 4 were representative. Adjacency matrices were then converted into edge lists, and visualized with igraph (*v 2.1.4*, (29)). To facilitate visualization, the edgelist was trimmed using an arbitrary hard threshold of 0.25, after testing a range of hard thresholds for representativeness. The circadian rhythm modules were excluded from the MEnetworks lest they drive major network structural changes that were simply due to different sampling times in the field.

Evaluation of the ME networks: To describe and compare ME network properties, we computed the number of edges (E) and network density (D; fraction of all possible module–module pairs connected), along with mean absolute edge weight $|w|$, and the percentage positive edges (%+E), where edge weights were signed adjacency values derived from pairwise ME correlations. To focus on the most informative connections and match network visualizations, E and D were calculated on thresholded edges ($|w|>0.25$), whereas |w| and %+E were calculated on the untrimmed signed adjacency matrix to summarize overall coupling independent of the hard-threshold choice.

Functional modularity of the network was evaluated using GO assortativity (Δfrac*W*), defined as the observed fraction of strong-edge absolute weight occurring within GO categories minus the mean of this quantity under a null generated by permuting GO category labels across modules while holding network topology and edge weights fixed. Significance was evaluated using a one-sided permutation test for enrichment and Benjamini–Hochberg correction across networks.

Module preservation analysis: Topological preservation of each module was formally calculated by building a multi-expression dataset using common genes within each module between laboratory and wild expression data and running the modulePreservation function with the laboratory network set as reference, and 200 rounds of permutations. Z-summary statistics were used to evaluate preservation status of each module.

Hub-gene network analysis

Hub gene identification: Hub genes were defined as genes with the highest 10% of within-module connectivity (kIN) in either the laboratory or wild-projected networks. kIN was calculated as the sum of adjacency values only within the module, and total connectivity (kTOT) was calculated as the row-sums of the adjacency matrices. Between-module connectivity was calculated as the difference between kTOT and kIN. Hub gene sets were obtained separately for each reference network (untreated, abiotic, biotic) and, where applicable, for wild-projected networks (spring and fall) using the same module assignments as for the laboratory networks.

Hub-gene network construction: Networks were generated to focus on the strongest connectivity relationships among intra-modular and inter-modular hub genes. For each network, the corresponding adjacency matrix was first subset to include only the entries between hub genes. To sparsify the graph while retaining strong connections, adjacency values in the sub-matrix were subjected to an upper quantile cutoff of 0.95. Self-edges were removed and undirected edge lists were constructed from the remaining non-zero entries. Reverse duplicates arising from the adjacency matrix symmetry were removed by sorting node pairs and discarding duplicated pairs. Graph objects using the package network *(v1.19.0,* (30)*)* were built from the filtered undirected edge list. The nodes were annotated with module membership for each gene based on the module–gene association tables and the edges with weights corresponding to the retained adjacency values. For visualization, we used the packages intergraph *(v2.0-4*, (31)*),* GGally *(v2.4.0,* (32)*)* and ggplot2 *(v4.0.1*, (33)*)*. Edge colors were discretized into three bins spanning the observed weight range (light to dark grey). Modules were sorted and colored by manually curated GO categories (Dataset S11) and arranged on a circle. Each module's hub genes were visualized around a position on the circle using a force-directed layout. Two levels of labelled convex hull annotations were added using ggplot's geom_mark_hall – colored hulls grouping modules by GO category and grey hulls around individual modules.

Random forest prediction of field scores

We used random forest regression models from the scikit-learn Python package *(v1.3.2,* (34)*)* to evaluate the predictive ability of MEs for phenotypical or metagenomic traits.

Feature normalization was applied in the first step using QuantileTransformer from the scikit-learn package. Uniform distribution and quantile number = 100 were used as parameters. For split season experiments, normalizations were applied separately per season. Pearson correlation (*r)* and R^2 score were used as measures of predictability. Permutation feature importance was used as a measure of the contribution for individual MEs. Monte Carlo cross-validation with a random 70% train/30% test split with 1,000 iterations was performed to generate distributions of *r*, R^2 scores and feature importances. Null distributions were created by randomly shuffling the trait values in each train/test split, then training and evaluating a random forest regression model on these datasets.

Mann-Whitney U tests (one-sided, "greater") were used to evaluate statistical significance, with Benjamini/Hochberg adjustment for multiple tests.

Analysis of general non-self response (GNSR) genes

Hellinger-transformed expression values were subset to contain only the 24 GNSR genes (35). Euclidean distance was calculated including either the full gene list, only the 24 GNSR genes, or 1,000 bootstraps of 24 randomly chosen genes. Variance explained by different factors was estimated using PERMANOVA (adonis2), where R^2 represents the fraction of total multivariate variance in transcriptomic distances attributable uniquely to that factor. Significance was determined via 999 permutations.

All analyses were performed in the R programming environment (*v4.4.0*, (36)), unless otherwise specified.

Figures

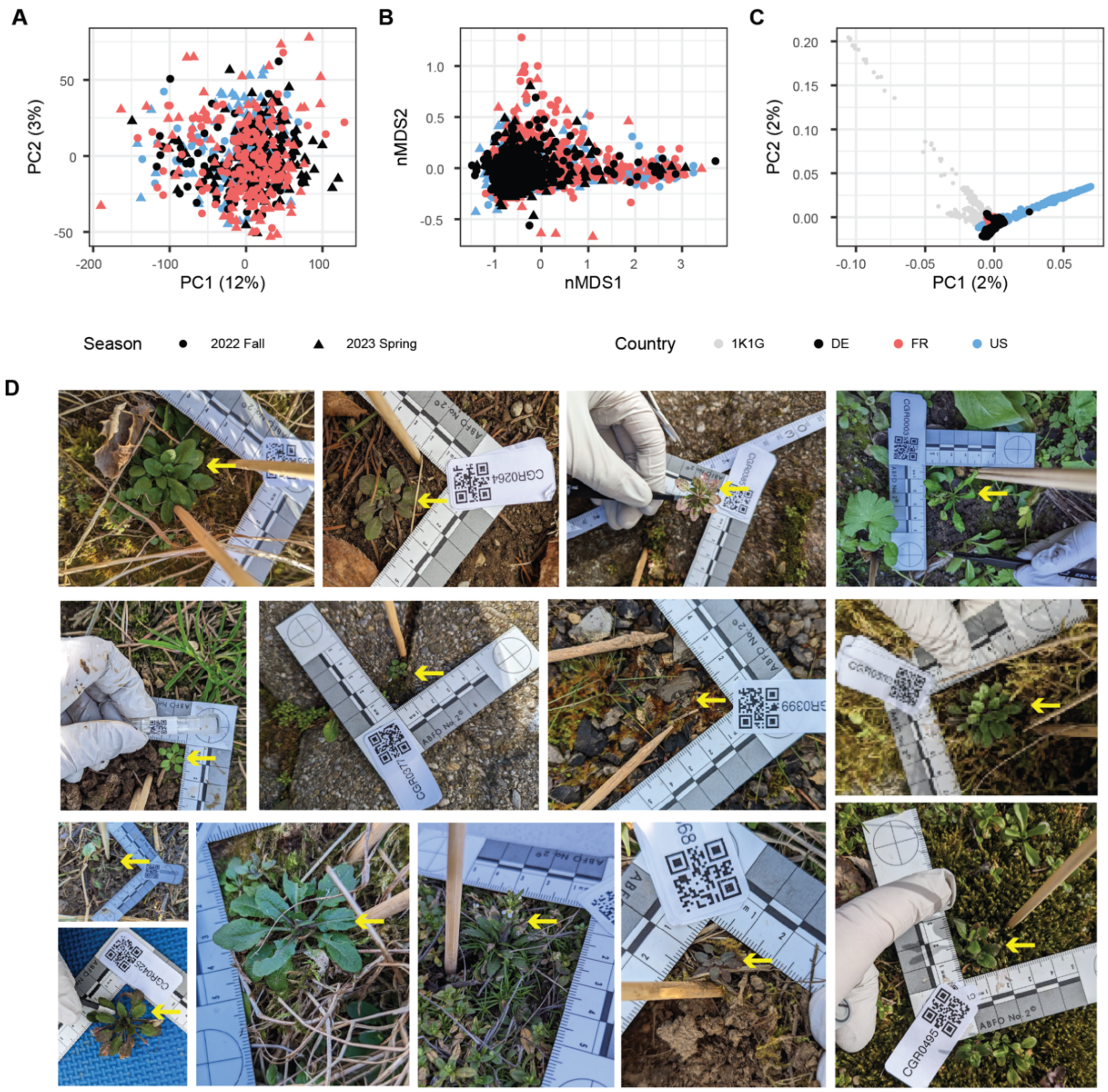

Fig. S1.1. Overview of A) transcriptomic, B) metagenomic, C) host genetic, and D) host phenotypic variation among samples. While host genetics is structured by sampling location, transcriptomes and microbiomes are not structured by either country or season. Yellow arrows in the field pictures point to *A. thaliana* plants sampled at different field sites.

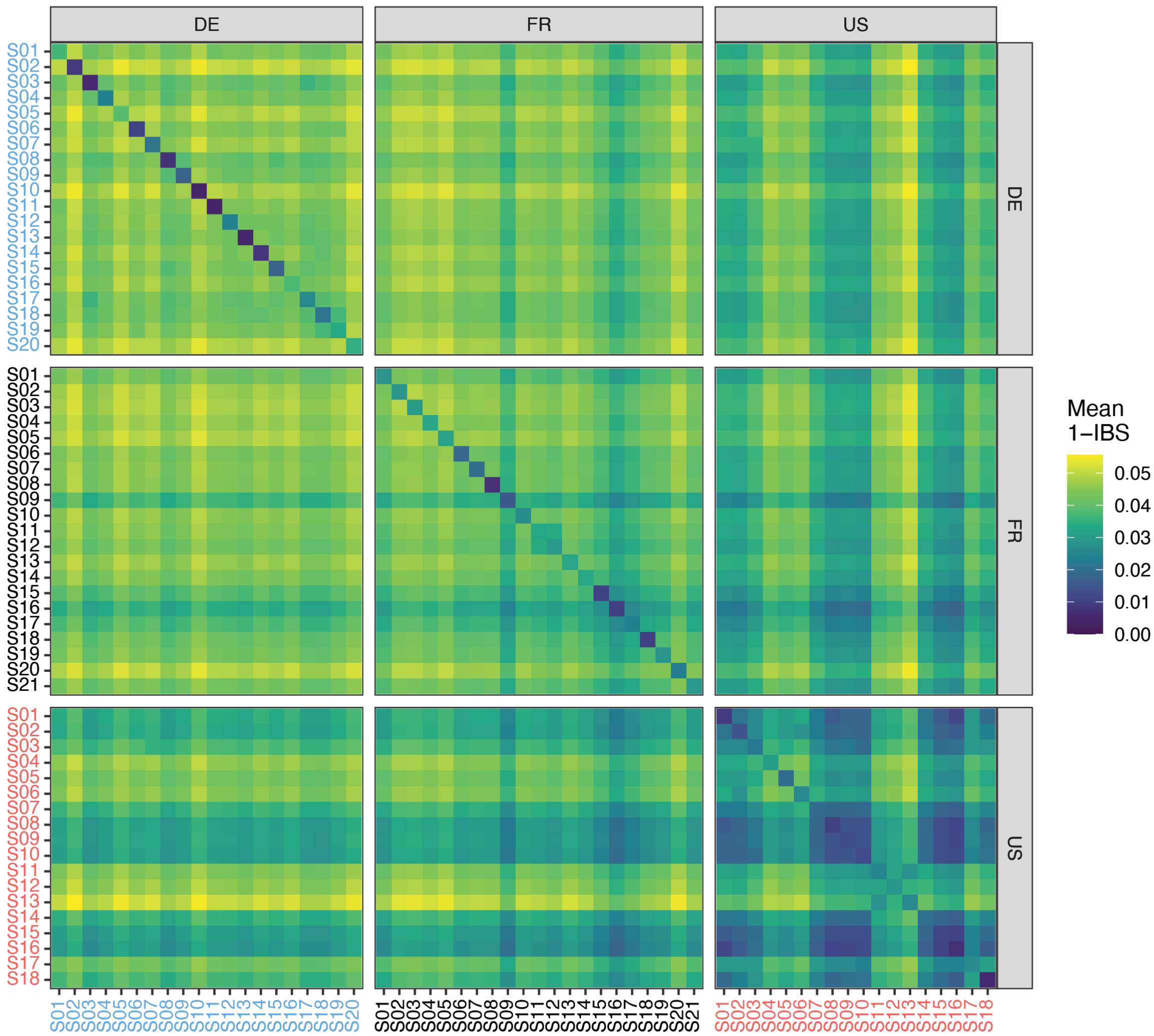

Fig. S1.2. Genetic diversity (measured as one minus Identity-by-State [IBS]) within and between countries. Enlarged version of Main Figure 1C. Each colored square illustrates the mean genetic distance (1-IBS) of all individuals within (on diagonal) or between sites. Sites are aggregated by countries, for correspondence between site code and location see https://doi.org/10.6084/m9.figshare.31638136.

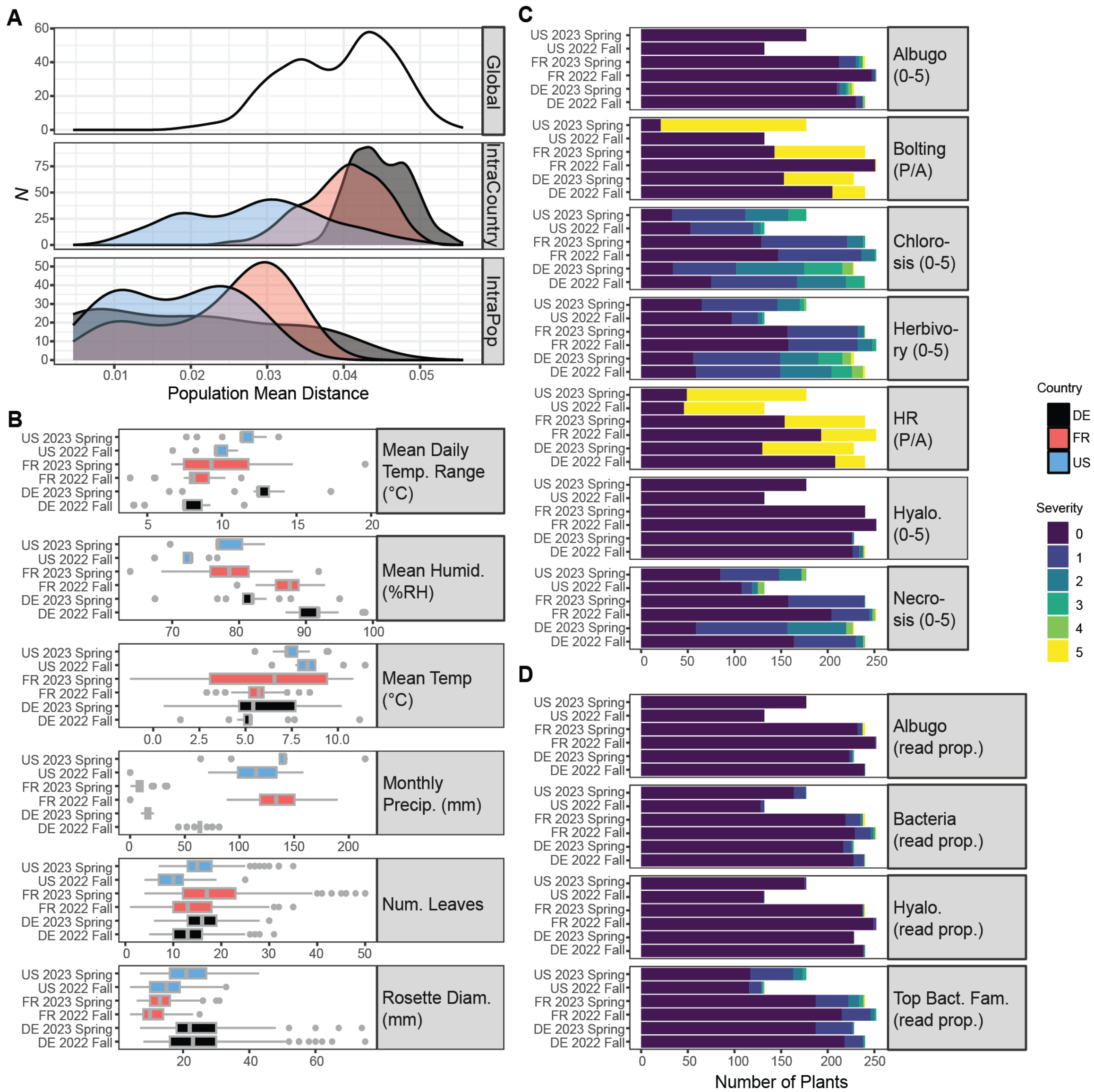

Fig. S2.1. Distribution of genetic, developmental, and disease characters across all plants. A) Histograms of inter-sample distances for plant pairs within sites (IntraPop), within countries but between sites (IntraCountry) and between countries (Global). For DE/FR, intra site distances are considerably smaller than inter-site, while USA has generally lower diversity. B) Distribution of weather and plant growth among countries and seasons. While we made efforts to ensure collections occurred at comparable climatic conditions and developmental stages between countries, some differences between countries remain, due to seasonal variation in weather over the growing season. C) Distribution of disease scores across all plants, by country and season. Chlorosis, necrosis, and HR-like symptoms are relatively common, especially in Europe in Spring. Oomycete disease is uncommon in Europe and exceptionally rare in USA. Herbivory was common in all countries and seasons, and especially so in Germany. X-axis depicts the Number of Plants. D) Microbial load of *Albugo*, *Hyaloperonospora*, all Bacteria, the most abundant Bacterial family per plant, reported as binned host-adjusted proportions (microbial reads/host reads). These results broadly reflect the field disease scores from C, highlight that bacterial disease is uncommon and oomycete disease is rare in these samples.

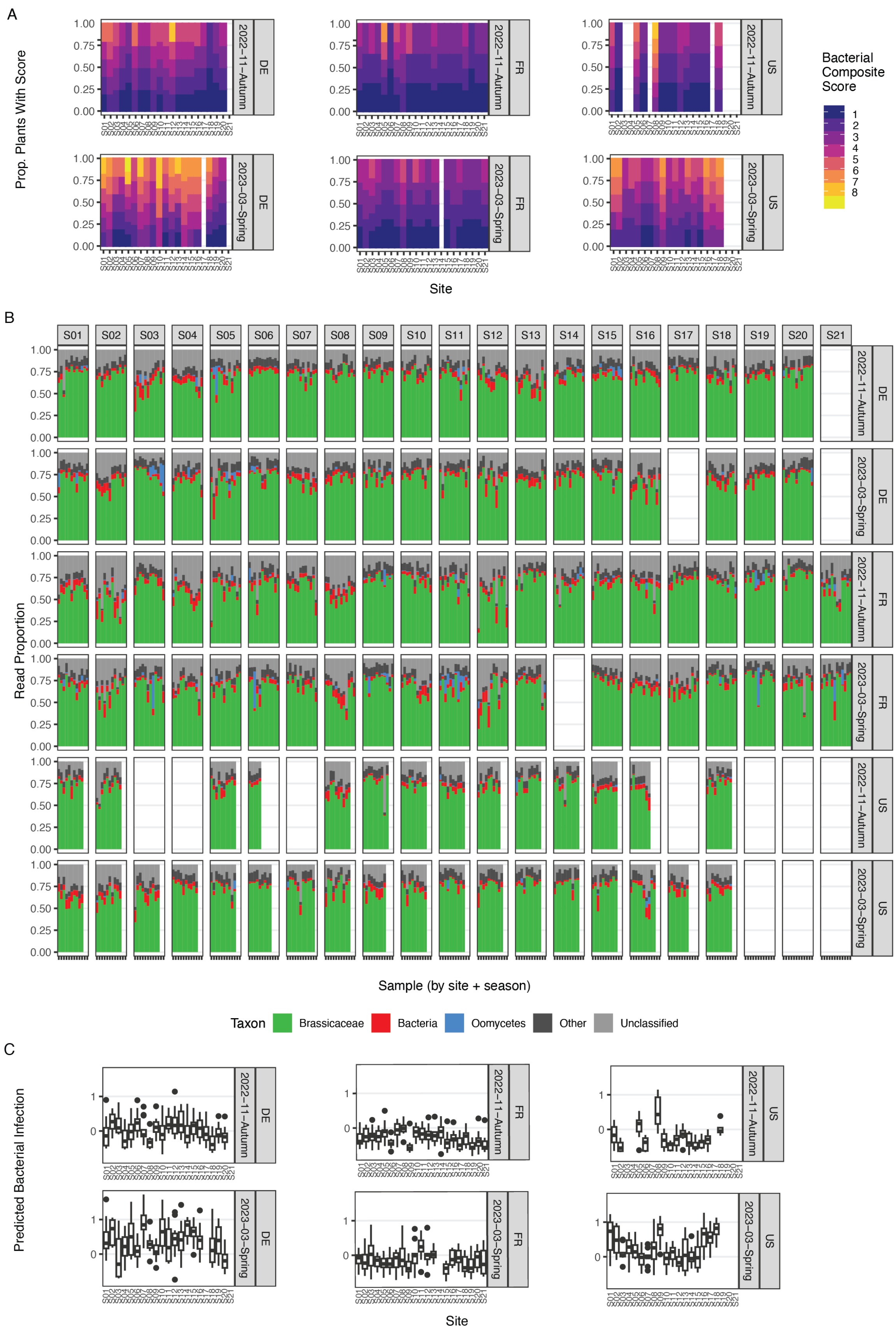
A
Prop. Plants With Score
2022−11−Autumn
2023−03−Spring
DE
FR
US
Site
Bacterial Composite Score
1
2
3
4
5
6
7
8
B
Read Proportion
Sample (by site + season)
Taxon
Brassicaceae
Bacteria
Oomycetes
Other
Unclassified
C
Predicted Bacterial Infection
Site

Fig. S2.2. Disease scores and metagenomic profiles of plant samples, grouped by country and season. A) Composite bacterial infection scores, calculated as the sum of symptoms suggestive of bacterial infection. Each vertical bar represents one site, and stacked colors indicate the proportion of individuals at that site assigned to each score. Symptoms varied among sites, countries, and seasons, with the fewest symptoms observed in France, and spring generally having higher pathogenesis scores than autumn. B) Microbial load and composition inferred from metagenomic sequencing. Each vertical bar represents one sampled individual, grouped by site and season. Stacked colors indicate the proportion of reads assigned to *Brassicaceae*, bacteria, oomycetes, other taxa, or unclassified reads; the non-*Brassicaceae* fraction provides a proxy for total microbial load including reads containing uncharacterized or taxonomically ambiguous sequences. The most abundant assigned microbial taxa included *Pseudomonas*, *Sphingomonas*, *Hyaloperonospora*, and *Albugo*. C) Latent estimates of bacterial infection inferred by confirmatory factor analysis. Each box plot shows the distribution of latent values within one site. Throughout, site labels correspond to those in https://doi.org/10.6084/m9.figshare.31638136, and plants (where listed) are in lexographic order.

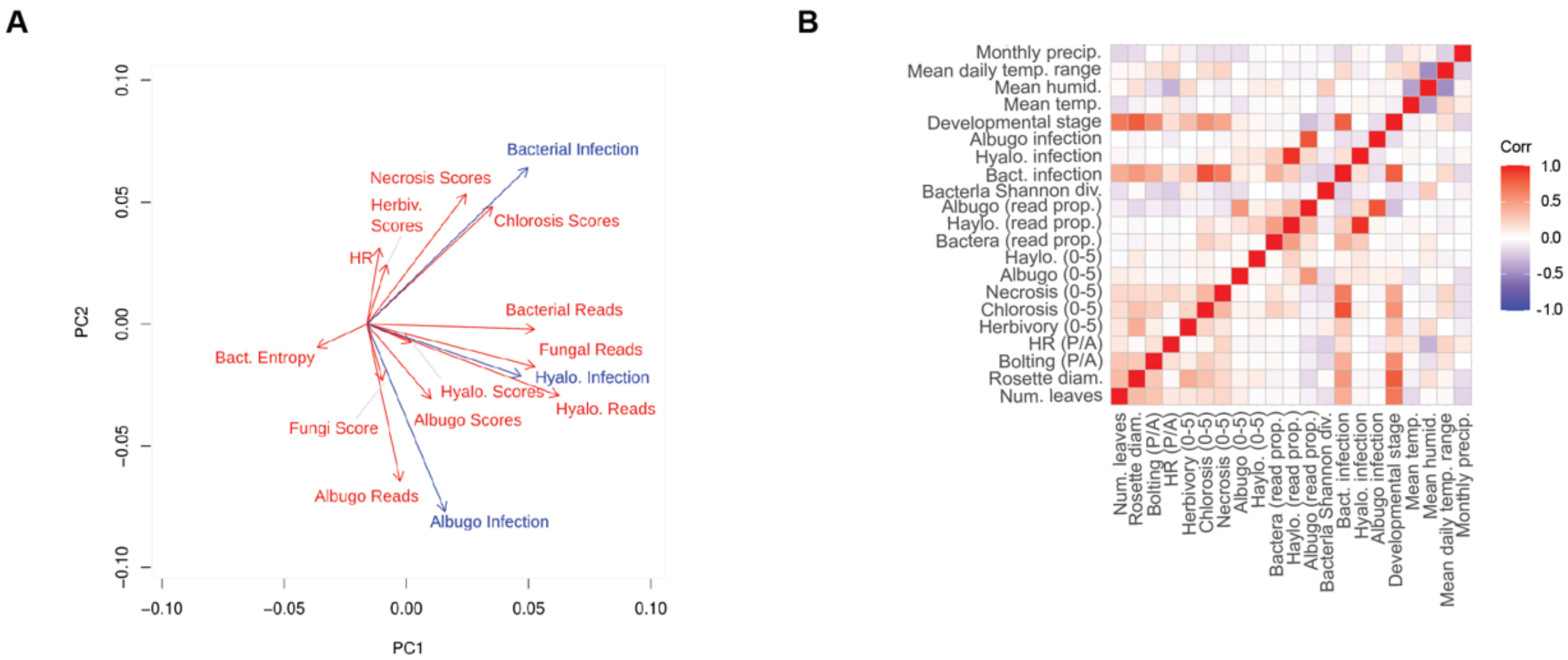

Fig. S3. Confirmatory factor analysis to synthesize latent variable representations of bacterial, *Hyaloperonospora* and *Albugo* infections from multiple imprecise observations. A) PCA highlights correlations among partially redundant measures of microbial infection. Latent estimates of infection (blue) integrated across these multiple predictors to form simple estimates of infection. B) Correlation among weather, microbial load, and plant disease scores and size. *Albugo* scores and load are highly positively correlated, while *Hyaloperonospora* and bacterial loads have weaker positive correlations.

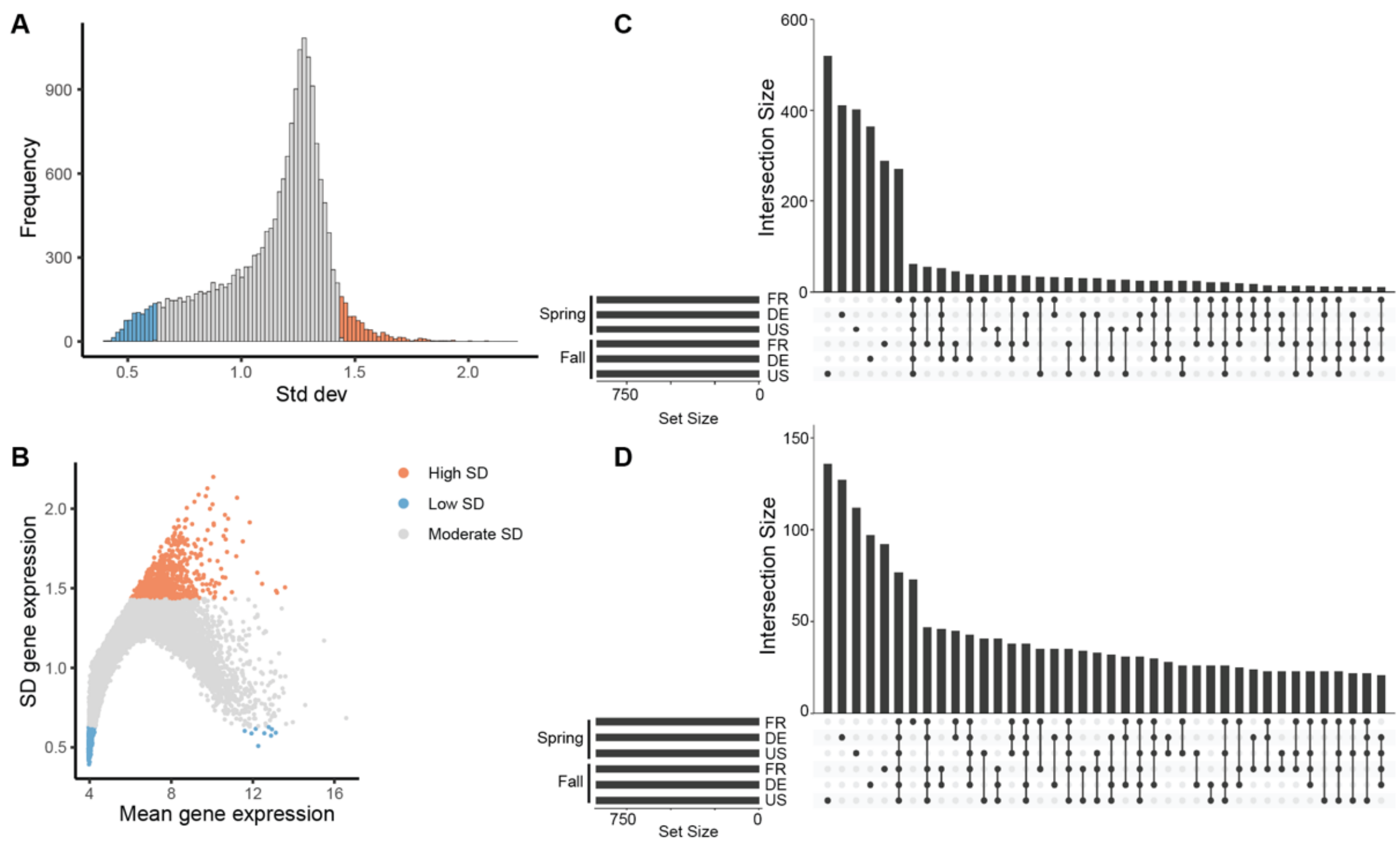

Fig. S4. Characterization of high- and low-variability (SD) genes. A) Histogram of standard deviation of all genes passing initial dropout and variance filter. Genes ranking top and bottom 5% of SD was identified as high- and low-SD genes (N=922 each). B) Relationships between average gene expression level and its SD. Note that while low-SD genes occupy expression extremes, high SD genes all have moderate expression levels. C-D) Upset plot showing gene sharing among high (panel C) and low (panel D) SD genes. High- and low-SD genes were called separately for each study region and season. The great majority of high- and low-SD genes are private to each region and season, especially for high SD genes.

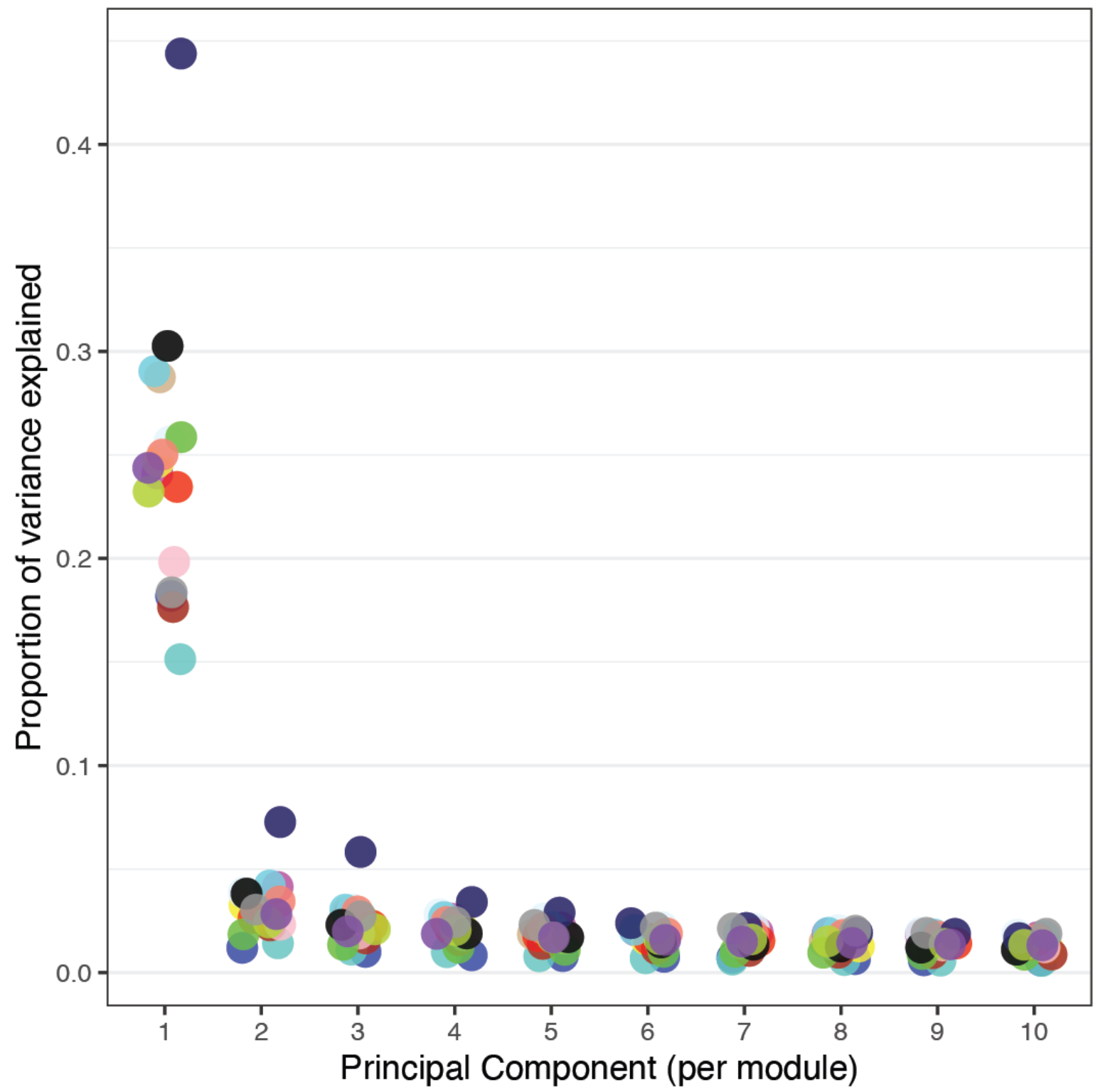

Fig. S5. Proportion of variance explained by the first 10 module PCs of the wild RNA-seq data. Each point represents one module, colored by their respective module identity. PC1 corresponds to the module eigengene (ME), which explained between 15-45% of the variance in respective modules.

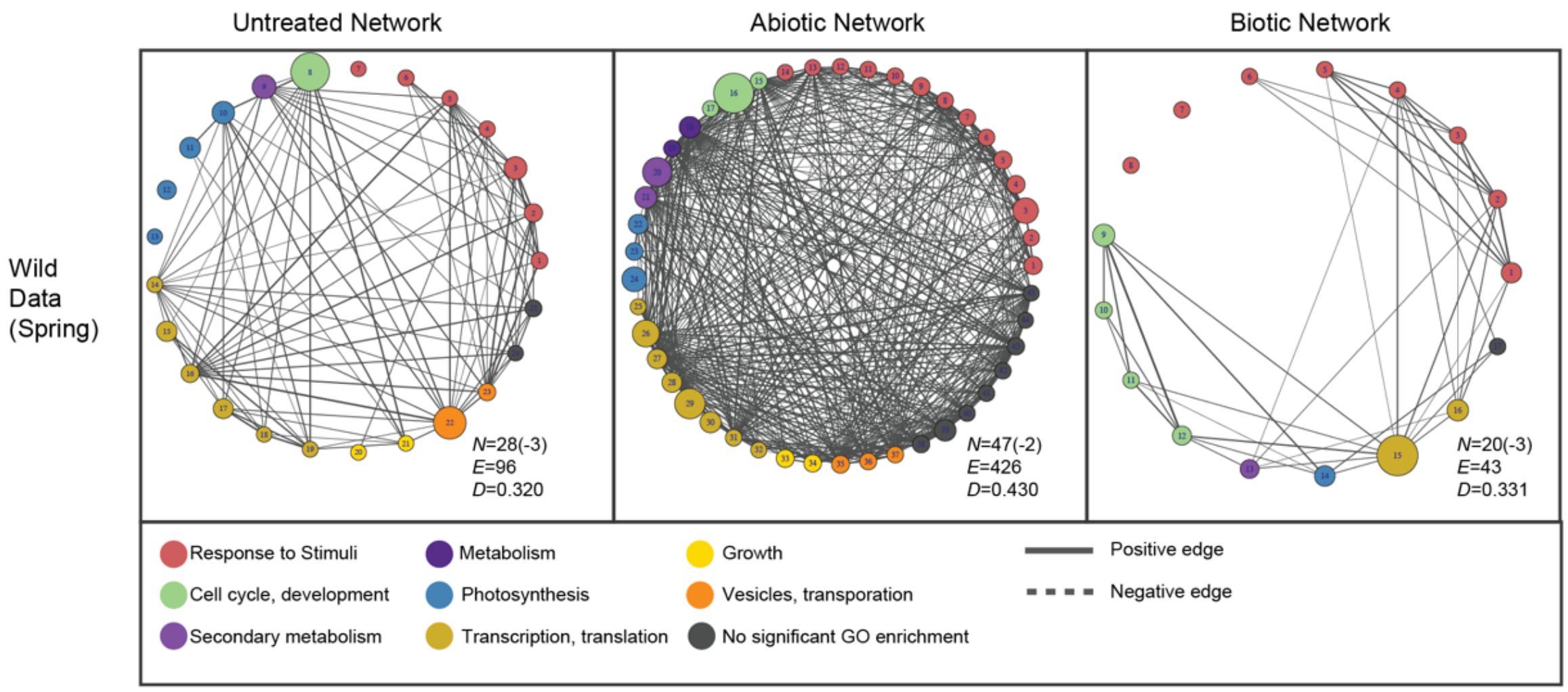

Fig. S6. Module eigengene (ME) networks of public laboratory modules projected to wild samples collected in spring 2023. Each filled bubble represents a module, colored: GO-enrichment categories, size: module size, edge width: strength of connection. *N*: Number of nodes/modules (minus circadian modules). *E*: edges. *D*: network density. Consistent with Fig. 4A, while the biotic ME network remains largely conserved between laboratory and projected wild data, the topology of the untreated and abiotic ME networks is heavily disrupted.

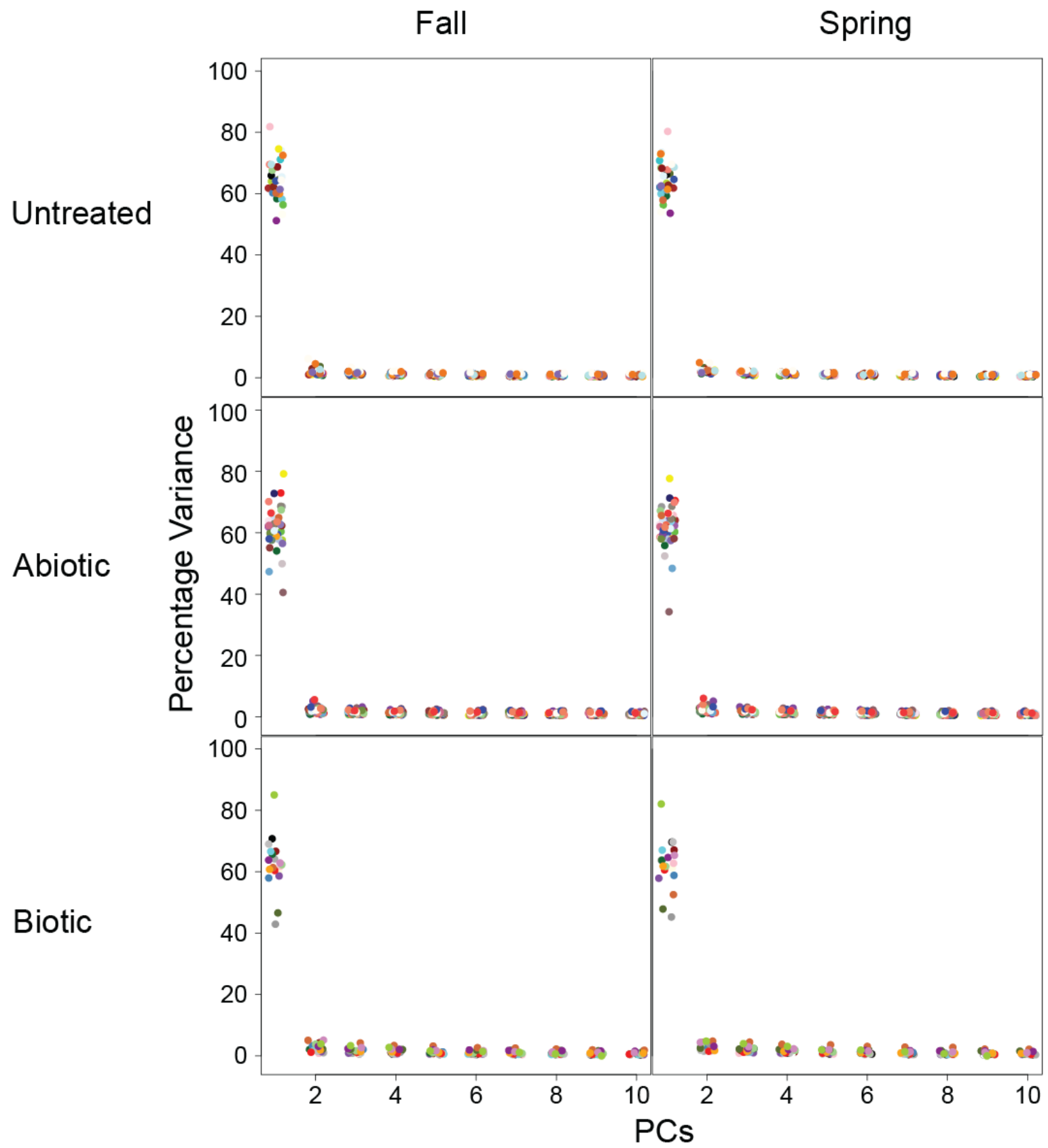

Fig. S7. Proportion of variance explained by the first 10 module PCs of the wild-projected laboratory WGCNs. Module assignments were acquired from independent WGCNA using published laboratory data of untreated (top), abiotic (middle), and biotic (bottom) treatment samples, and wild expression data from fall 2022 (left) and spring 2023 (right) were projected onto the modules. Each point represents one module, colored according to their designated color labels. PC1 corresponds to the module eigengene (ME), which explained an average of ~60% of the variance in respective modules.

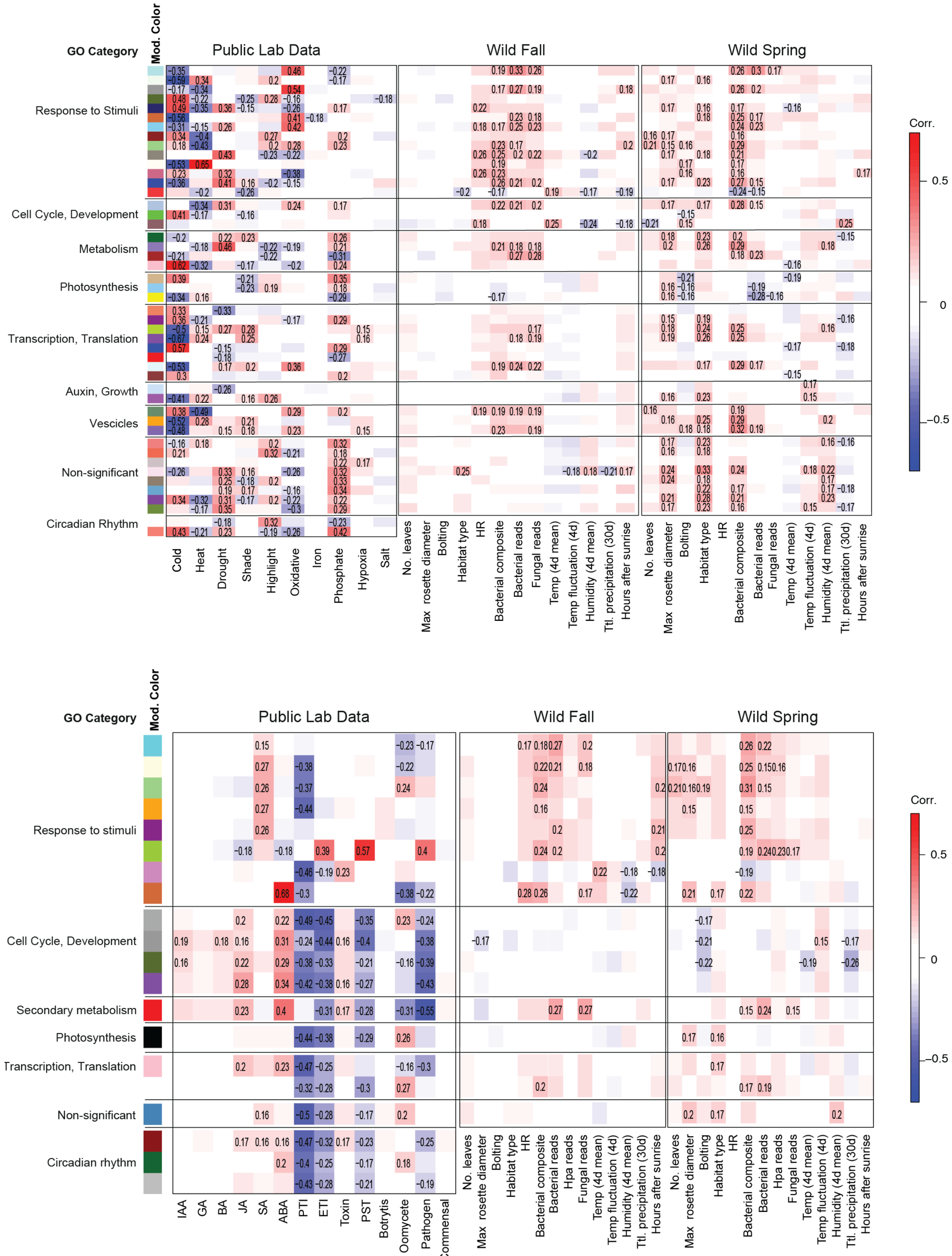

Fig. S8.1. Abiotic module–trait correlation heatmap. Pearson's correlation coefficients between eigengenes (MEs) of the public abiotic WGCNA and laboratory treatments (left), and between eigengenes of wild fall (middle) and spring (right) transcriptomes projected onto the abiotic modules and their corresponding field scores. Module colors are indicated by rectangles on the left, and modules are grouped into eight GO categories. Numbers in the heatmaps show Pearson's *r* for

significant ME–trait associations only ($p < 0.005$). Laboratory MEs exhibit strong, bidirectional correlations with applied treatments, whereas associations between projected wild MEs and field scores are weaker but generally positive.

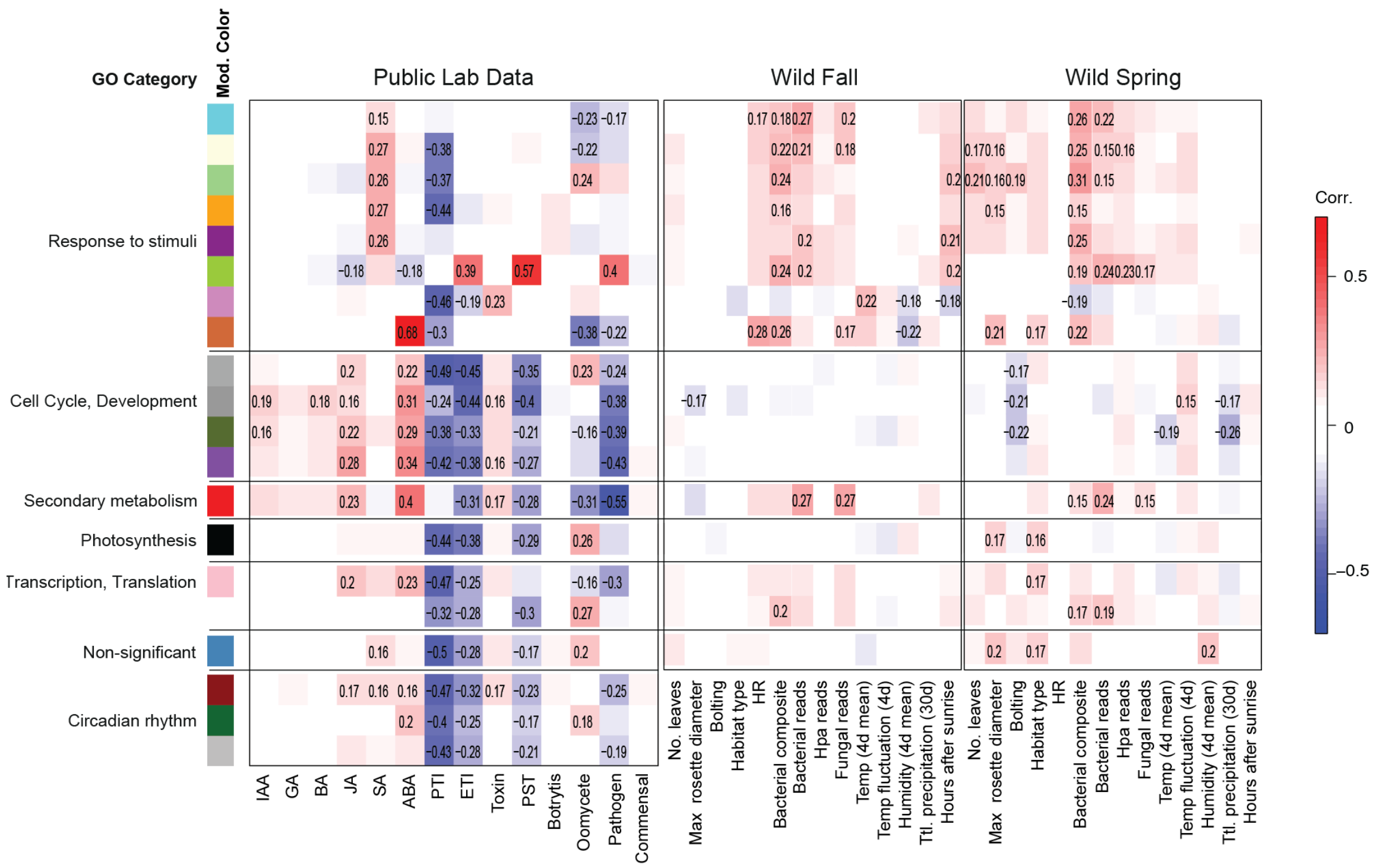

Fig. S8.2. Biotic module–trait correlation heatmap. Pearson's correlation coefficients between eigengenes (MEs) of the public biotic WGCNA and laboratory treatments (left), and between eigengenes of wild fall (middle) and spring (right) transcriptomes projected onto the biotic modules and their corresponding field scores. Module colors are indicated by rectangles on the left, and modules are grouped into six GO categories. Numbers in the heatmaps show Pearson's r for significant ME–trait associations only ($p < 0.005$). Laboratory MEs exhibit strong, bidirectional correlations with applied treatments, whereas associations between projected wild MEs and field scores are weaker but generally positive. The strong negative associations between laboratory infections and growth-related functions are most notably missing in the wild.

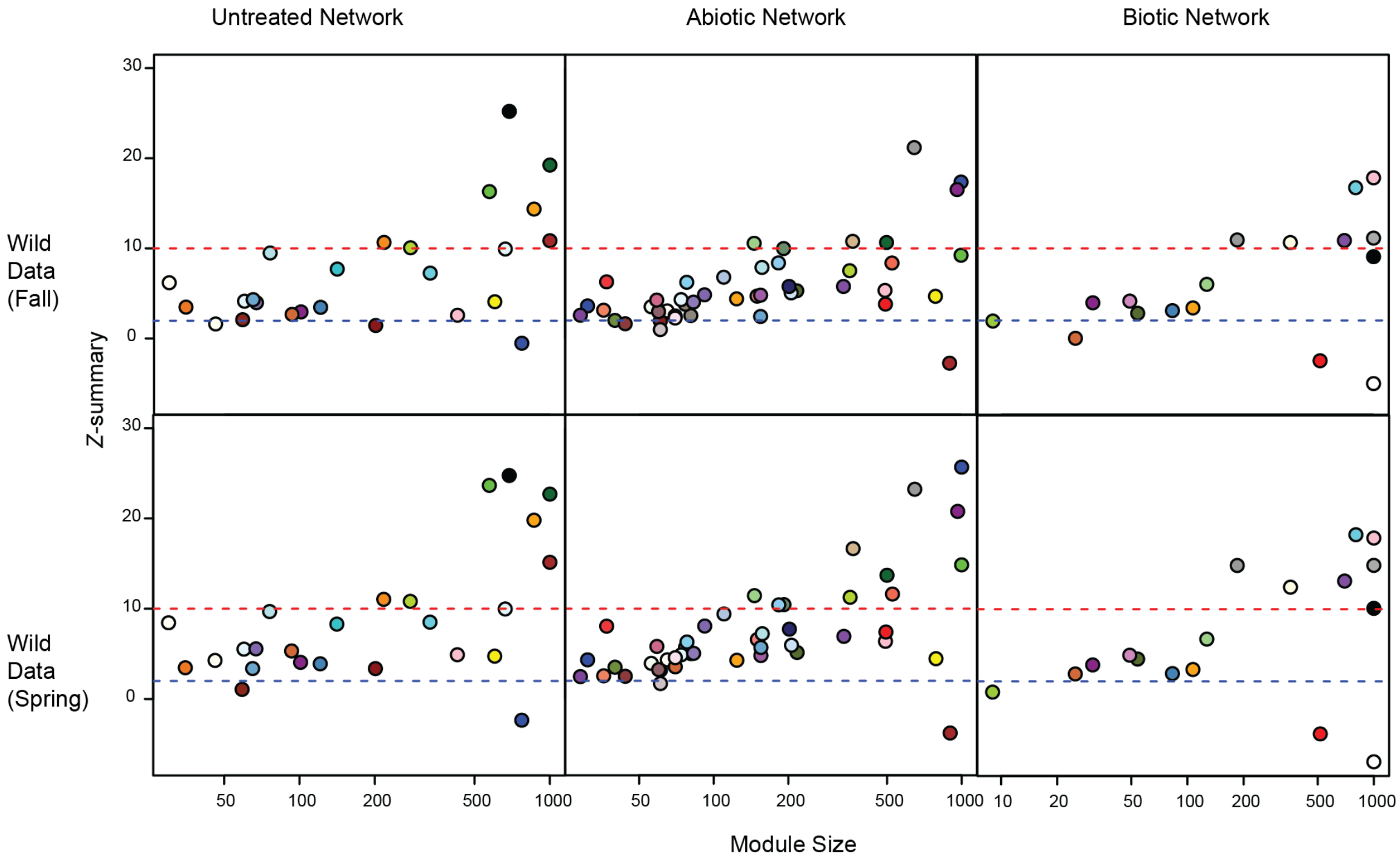

Fig. S9. Module preservation statistics of laboratory-derived WGCNs after the projection of wild fall (top panel) and spring (bottom panel) samples. Modules are colored according to designated color labels. *Z*-summary score>10: highly conserved, >2: moderately conserved, <2: unconserved. Both untreated and abiotic projections showed abundant moderately conserved modules, while biotic network has less middle category, and featured greater proportion of highly conserved modules.

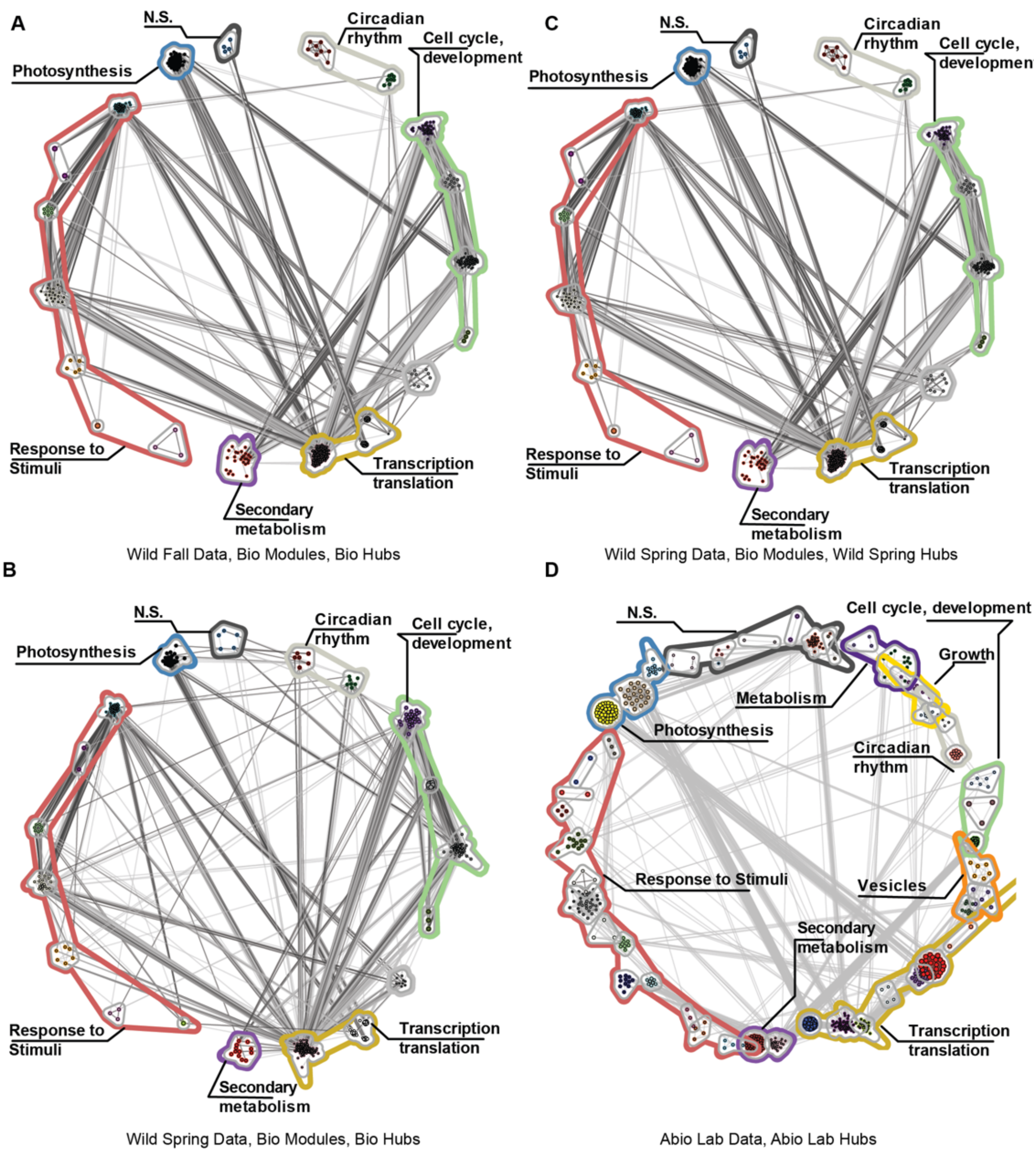

Fig. S10.1. Hub-gene networks from wild-projected biotic networks (A-C) and abiotic laboratory networks (D). A-B) Hub-gene networks constructed from hub genes (top 10% by within-module connectivity) identified in biotic networks projected from wild samples collected in fall 2022 (A) and spring 2023 (B). C) Network constructed from hub genes identified within the laboratory biotic network, with inter-gene relationships projected from wild samples collected in spring 2023. D) Hub-gene network of public abiotic data. Modules are grouped by GO enrichment categories, indicated by colored contours. Stronger connections between hub genes are shown by darker grey edges.

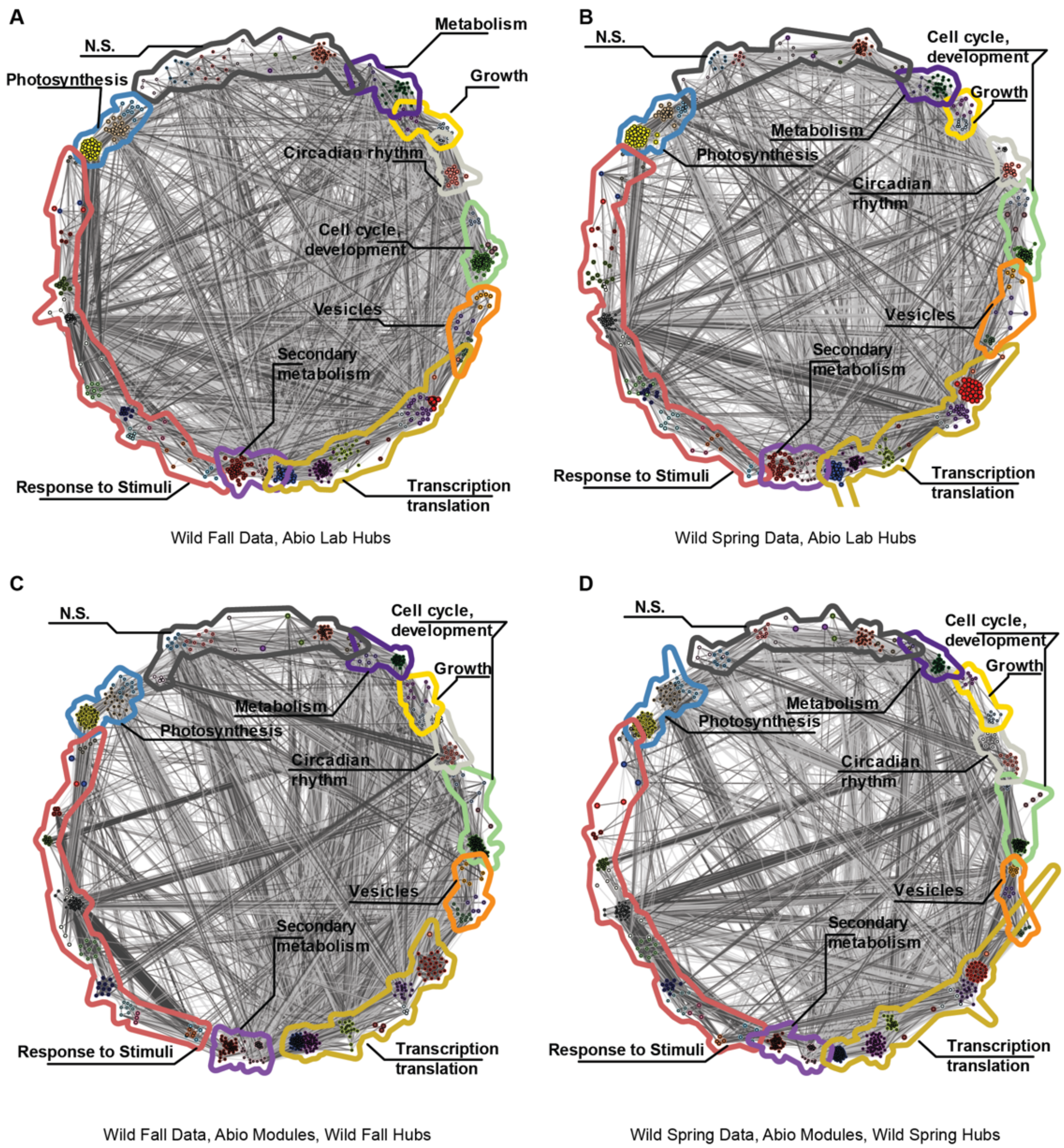

Fig. S10.2. Hub-gene networks from wild-projected abiotic networks. A-B) Hub-gene networks constructed from hub genes identified within the laboratory abiotic network, with inter-gene relationships projected from wild samples collected in fall 2022 (A) and spring 2023 (B). C-D) Networks constructed from hub genes identified in abiotic networks projected from wild samples collected in fall 2022 (C) and spring 2023 (D). Modules are grouped by GO enrichment categories, indicated by colored contours. Stronger connections between hub genes are shown by darker grey edges.

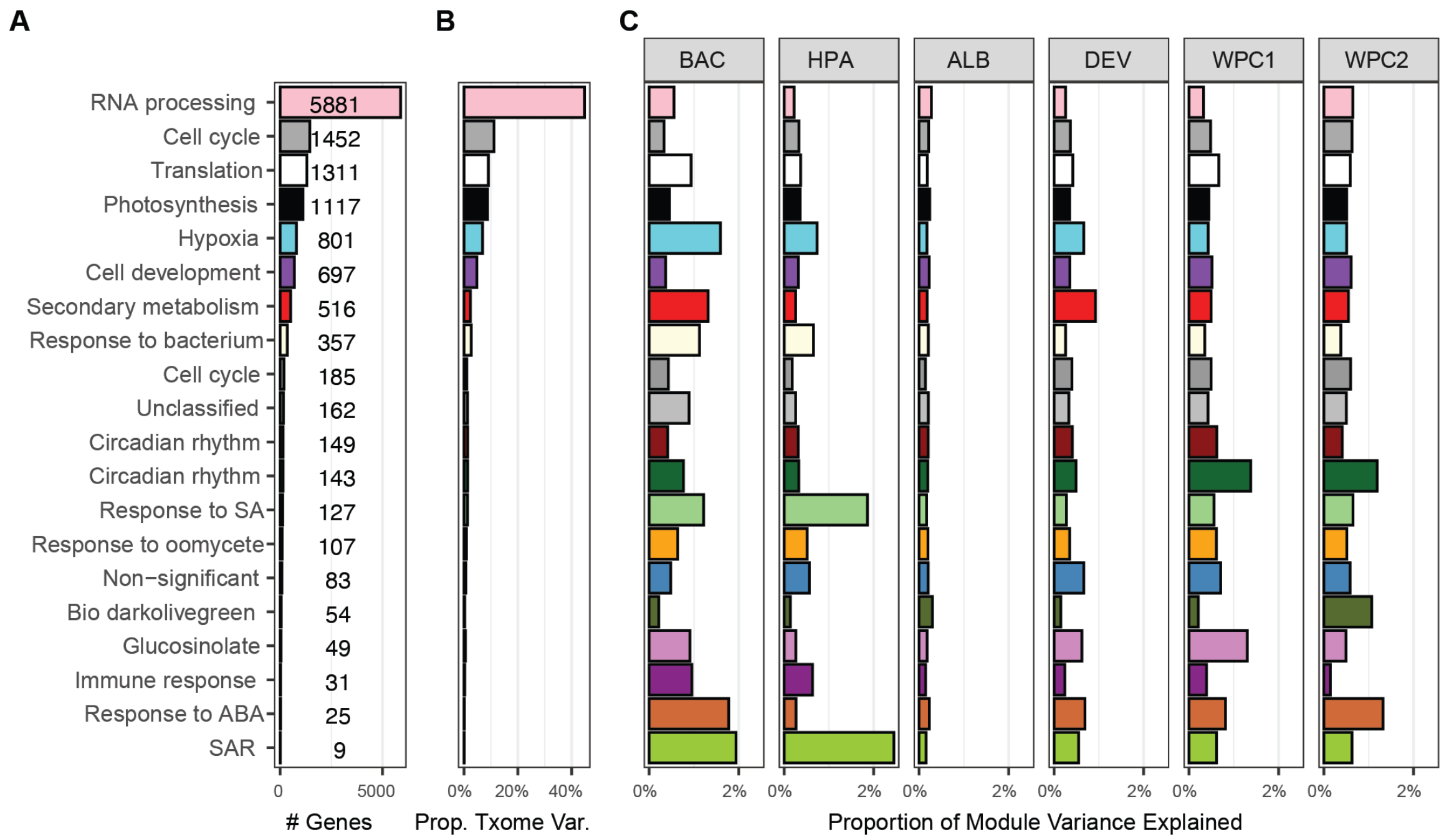

Fig. S11. RDA variance decomposition across biotic laboratory modules. A) Proportion of global transcriptome variance attributable to each of the projected biotic laboratory WCGNA modules, and number of genes in each module. B) Proportion of module expression variance explained by constraints in per-module RDA analyses, constrained by the same factors as main Fig 3. C) Proportion global module expression variance uniquely explained by each predictor.

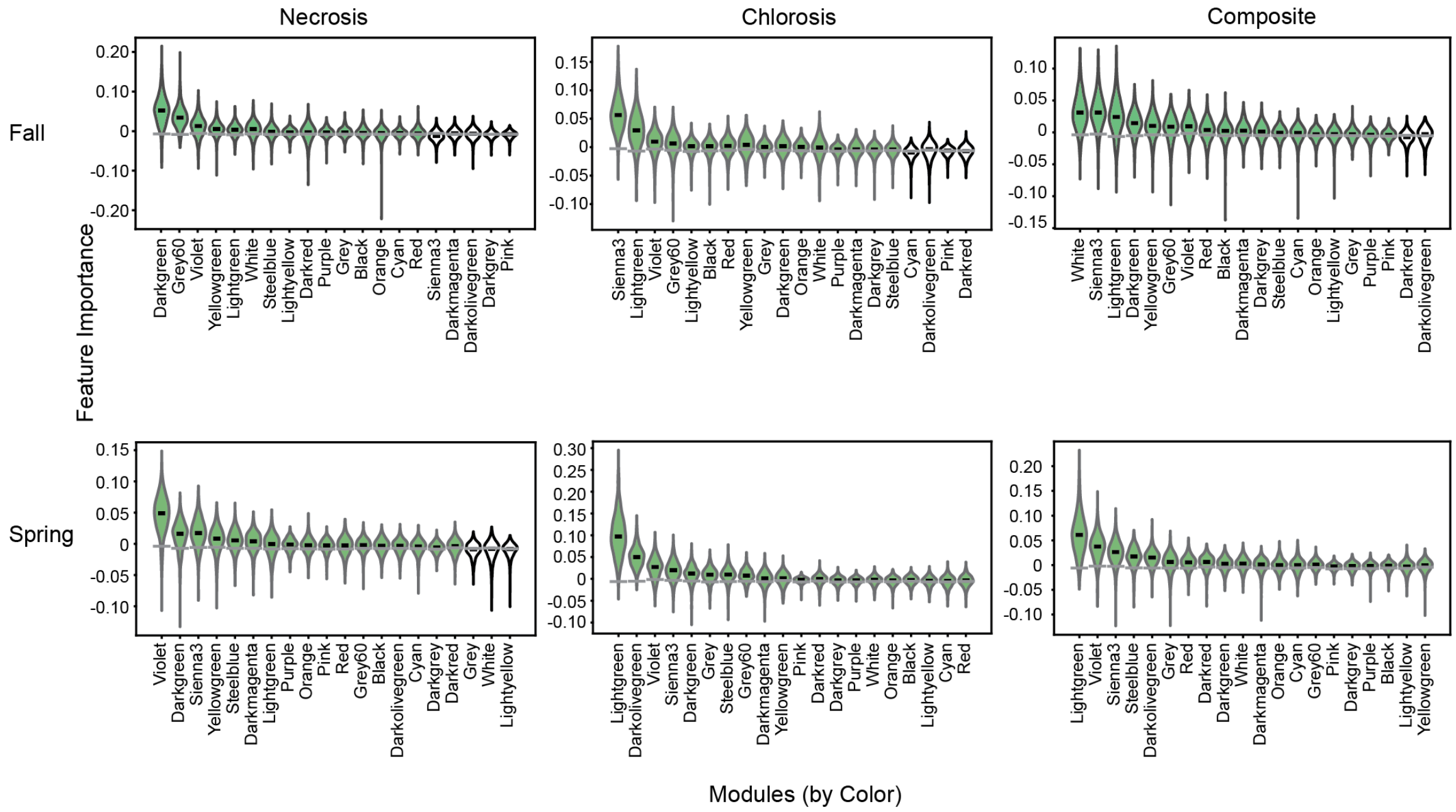

Fig. S12. Feature importance scores for each module eigengene (ME) in random forest predictions. Necrosis (left), chlorosis (middle), and their composite (right) from fall (top) and spring (bottom) data, respectively. MEs were calculated after projection of wild data onto public biotic modules. Each violin shows the distribution of module feature importance in 1000x random forest iterations, while the grey bars at the bottom of each violin illustrates the median of 1000x permuted null distribution.

SI references

1. H. Yaffe, *et al.*, LogSpin: a simple, economical and fast method for RNA isolation from infected or healthy plants and other eukaryotic tissues. *BMC Res. Notes* **5**, 45 (2012).
2. S. Picelli, *et al.*, Tn5 transposase and tagmentation procedures for massively scaled sequencing projects. *Genome Res.* **24**, 2033–2040 (2014).
3. W. Yuan, *et al.*, Pervasive under-dominance in gene expression underlying emergent growth trajectories in Arabidopsis thaliana hybrids. *Genome Biol.* **24**, 200 (2023).
4. K. D. Murray, J. O. Borevitz, D. Weigel, N. Warthmann, Acanthophis: a comprehensive plant hologenomics pipeline. *J. Open Source Softw.* **9**, 6062 (2024).
5. M. Schubert, S. Lindgreen, L. Orlando, AdapterRemoval v2: Rapid Adapter Trimming, Identification, and Read Merging. *BMC Res. Notes* **9**, 88 (2016).
6. P. Lamesch, *et al.*, The Arabidopsis Information Resource (TAIR): improved gene annotation and new tools. *Nucleic Acids Res.* **40**, D1202–10 (2012).
7. H. Li, Aligning Sequence Reads, Clone Sequences and Assembly Contigs with BWA-MEM. (2013).
8. T. Yun, *et al.*, Accurate, scalable cohort variant calls using DeepVariant and GLnexus. *Bioinformatics* **36**, 5582–5589 (2021).
9. R. Poplin, *et al.*, A universal SNP and small-indel variant caller using deep neural networks. *Nat. Biotechnol.* **36**, 983–987 (2018).
10. P. Wlodzimierz, *et al.*, Cycles of satellite and transposon evolution in Arabidopsis centromeres. *Nature* **618**, 557–565 (2023).
11. L. C. Teasdale, *et al.*, Pangenomic context reveals the extent of intraspecific plant NLR evolution. *Cell Host & Microbe* **33**, 1291–1305.e9 (2025).
12. Y. Bai, *et al.*, Functional overlap of the Arabidopsis leaf and root microbiota. *Nature* **528**, 364–369 (2015).
13. D. E. Wood, J. Lu, B. Langmead, Improved metagenomic analysis with Kraken 2. *Genome Biol.* **20**, 257 (2019).
14. Y. Rosseel, lavaan: An R Package for Structural Equation Modeling. *Journal of Statistical Software* (2012).
15. H. Li, Seqtk - Toolkit for Processing Sequences in FASTA/Q Formats. *GitHub* [Preprint] (2008). Available at: https://github.com/lh3/seqtk.
16. C.-Y. Cheng, *et al.*, Araport11: a complete reannotation of the Arabidopsis thaliana reference genome. *Plant J.* **89**, 789–804 (2017).
17. B. Li, C. N. Dewey, RSEM: accurate transcript quantification from RNA-Seq data with or without a reference genome. *BMC Bioinformatics* **12**, 323 (2011).
18. J. Köster, S. Rahmann, Snakemake --- a Scalable Bioinformatics Workflow Engine. *Bioinformatics* **28**, 2520–2522 (2012).
19. A. Conesa, *et al.*, A survey of best practices for RNA-seq data analysis. *Genome Biol.* **17**, 13 (2016).
20. M. I. Love, W. Huber, S. Anders, Moderated estimation of fold change and dispersion for RNA-seq data with DESeq2. *Genome Biol.* **15**, 550 (2014).
21. P. Dixon, VEGAN, a package of R functions for community ecology. *J. Veg. Sci.* **14**, 927–930 (2003).
22. G. K. Smyth, "limma: Linear Models for Microarray Data" in *Bioinformatics and Computational Biology Solutions Using R and Bioconductor*, (Springer-Verlag, 2005), pp. 397–420.
23. M. E. Ritchie, *et al.*, limma powers differential expression analyses for RNA-sequencing and microarray studies. *Nucleic Acids Res.* **43**, e47 (2015).
24. H. Mi, A. Muruganujan, D. Ebert, X. Huang, P. D. Thomas, PANTHER version 14: more genomes, a new PANTHER GO-slim and improvements in enrichment analysis tools. *Nucleic Acids Res.* **47**, D419–D426 (2019).
25. P. Langfelder, S. Horvath, WGCNA: an R package for weighted correlation network analysis. *BMC Bioinformatics* 9, 559 (2008).
26. Y. Yu, H. Zhang, Y. Long, Y. Shu, J. Zhai, Plant Public RNA-seq Database: a comprehensive online database for expression analysis of ~45 000 plant public RNA-Seq libraries. *Plant Biotechnol. J.* 20, 806–808 (2022).

27. G. Yu, Thirteen years of clusterProfiler. *Innovation (Camb.)* **5**, 100722 (2024).
28. M. Carlson, org.At.tair.db: Genome wide annotation for Arabidopsis. [Preprint] (2024).
29. G. Csárdi, *et al.*, igraph: Network Analysis and Visualization in R. [Preprint] (2026). Available at: http://dx.doi.org/10.5281/zenodo.7682609.
30. C. T. Butts, network: A Package for Managing Relational Data in R. *J. Stat. Softw.* **24**, 1–36 (2008).
31. M. Bojanowski, Intergraph: Coercion Routines for Network Data Objects. (2023). Available at: https://mbojan.github.io/intergraph/ [Accessed 26 February 2026].
32. D. Cook, *et al.*, GGally: Extension to “ggplot2.” *Comprehensive R Archive Network (CRAN)* (2025). Available at: https://CRAN.R-project.org/package=GGally [Accessed 26 February 2026].
33. H. Wickham, *Ggplot2*, 2nd Ed. (Springer International Publishing, 2016).
34. F. Pedregosa, *et al.*, Scikit-learn: Machine Learning in Python. *J. Mach. Learn. Res.* **12**, 2825–2830 (2011).
35. B. A. Maier, et al., A general non-self response as part of plant immunity. *Nat Plants* 7, 696–705 (2021).
36. R Core Team, R: A Language and Environment for Statistical Computing. [Preprint] (2024). Available at: https://www.R-project.org/.

TEAM PATHOCOM Authors

The following authors were part of the TEAM PATHOCOM

Haim Ashkenazy[1], Tien-Hao Chang[2], Choghag Demijirian[2], Rémi Duflos[3], Alejandra Duque-Jaramillo[1], Katrin Fritschi[1], Andrew Gloss[2], Anette Habring[1], Katharina Klein[2], Eric Laderman[2], Irene Leccia[3], Roxane Lion[3], Hanna Märkle[2], Baptiste Mayjonade[3], Timothy C. Morton[2], Ankita Pandey[2], Alexandre Perrat[3], Kamal Radharamanan[2], Angelique Rat[3], Sonia Silva[3], Manmeet Singh[2], Daniel de Souza[2], Hannah Whitehurst[2]

[1]Department of Molecular Biology, Max Planck Institute for Biology Tübingen, 72076 Tübingen
[2]Department of Biology, New York University, New York, NY 10003, USA
[3]LIPME, CNRS, INRAE, Université de Toulouse, 31326 Castanet Tolosan, France

Author contributions
TEAM PATHOCOM assisted with field collections and laboratory work and contributed advice.
