# Supplementary material for: Biotic response networks are an important organizer of the transcriptome in wild Arabidopsis thaliana populations
Source: Proc Natl Acad Sci U S A. 2026 Oct 19;123(43):e2611539123. doi: 10.1073/pnas.2611539123 (PMC13715808; doi:10.1073/pnas.2611539123)
Supplement: Supplementary file 2 [file pnas.2611539123.sd15.docx]

**TEAM PATHOCOM Authors**

The following authors were part of the TEAM PATHOCOM

Haim Ashkenazy^1^, Tien-Hao Chang^2^, Choghag Demijirian^2^, Rémi Duflos^3^, Alejandra Duque-Jaramillo^1^, Katrin Fritschi^1^, Andrew Gloss^2^, Anette Habring^1^, Katharina Klein^2^, Eric Laderman^2^, Irene Leccia^3^, Roxane Lion^3^, Hanna Märkle^2^, Baptiste Mayjonade^3^, Timothy C. Morton^2^, Ankita Pandey^2^, Alexandre Perrat^3^, Kamal Radharamanan^2^, Angelique Rat^3^, Sonia Silva^3^, Manmeet Singh^2^, Daniel de Souza^2^, Hannah Whitehurst^2^

^1^Department of Molecular Biology, Max Planck Institute for Biology Tübingen, 72076 Tübingen

^2^Department of Biology, New York University, New York, NY 10003, USA

^3^LIPME, CNRS, INRAE, Université de Toulouse, 31326 Castanet Tolosan, France

**Author contributions**

TEAM PATHOCOM assisted with field collections and laboratory work and contributed advice.
